# Social rights, public policies, and social indicators related to persons with disabilities in Brazil: a scoping review

**DOI:** 10.1590/S2237-96222026v35e20250679.en

**Published:** 2026-07-22

**Authors:** Cristina Maria Rabelais Duarte, Dolores Maria Franco de Abreu, Mariana Gabriel, Silvia de Oliveira Pereira, Maria Fernanda de Faria von Sydow Bittencourt, Daniele da Silva Garcez Novaes, Maritsa Carla de Bortoli

**Affiliations:** 1 Fundação Oswaldo Cruz, Núcleo de Informação, Política Pública e Inclusão Social, Rio de Janeiro, RJ, Brasil. Fundação Oswaldo Cruz Fundação Oswaldo Cruz Núcleo de Informação, Política Pública e Inclusão Social Rio de Janeiro RJ Brazil; 2 Fundação Oswaldo Cruz, Núcleo de Informação, Política Pública e Inclusão Social, Rio de Janeiro, RJ, Brasil. Fundação Oswaldo Cruz Fundação Oswaldo Cruz Núcleo de Informação, Política Pública e Inclusão Social Rio de Janeiro RJ Brazil; 3 Fundação Oswaldo Cruz, Núcleo de Informação, Política Pública e Inclusão Social, Rio de Janeiro, RJ, Brasil. Fundação Oswaldo Cruz Fundação Oswaldo Cruz Núcleo de Informação, Política Pública e Inclusão Social Rio de Janeiro RJ Brazil; 4 Fundação Oswaldo Cruz, Núcleo de Informação, Política Pública e Inclusão Social, Rio de Janeiro, RJ, Brasil. Fundação Oswaldo Cruz Fundação Oswaldo Cruz Núcleo de Informação, Política Pública e Inclusão Social Rio de Janeiro RJ Brazil; 5 Centro Universitário UNIFASE, Núcleo de Informação, Política Pública e Inclusão Social, Rio de Janeiro, RJ, Brasil. Centro Universitário UNIFASE Núcleo de Informação, Política Pública e Inclusão Social Rio de Janeiro RJ Brazil; 6 Fundação Oswaldo Cruz, Núcleo de Informação, Política Pública e Inclusão Social, Rio de Janeiro, RJ, Brasil. Fundação Oswaldo Cruz Fundação Oswaldo Cruz Núcleo de Informação, Política Pública e Inclusão Social Rio de Janeiro RJ Brazil; 7 Fundação Oswaldo Cruz, Núcleo de Informação, Política Pública e Inclusão Social, Rio de Janeiro, RJ, Brasil. Fundação Oswaldo Cruz Fundação Oswaldo Cruz Núcleo de Informação, Política Pública e Inclusão Social Rio de Janeiro RJ Brazil

**Keywords:** Persons with Disabilities, Public Policy, Social Indicators, Scoping Review, Social Inclusion., Personas con Discapacidad, Política Pública, Indicadores Sociales, Revisión de Alcance, Inclusión Social.

## Abstract

Objective: To map the scientific evidence on social rights, public policies, and social indicators aimed at persons with disabilities in Brazil, identifying knowledge gaps, methodological trends, and opportunities to improve inclusive public policies.

Methods: Studies published from 2007 to April 2025 were included, with no restrictions regarding language or publication status, encompassing scientific articles, dissertations, theses, primary and secondary studies, theoretical and documentary analyses, case studies, policy evaluations, reports, and statistical yearbooks. Study selection and data extraction were performed by four independent reviewers using the Rayyan platform and, subsequently, Excel spreadsheets. Studies addressing rights, public policies, and indicators related to persons with disabilities, across different territorial scales, were included. Data were presented narratively.

Results: Of the 1,392 studies identified, 124 were included. Although the social model of disability was cited, the approach centered on the “characteristics of disability” predominated, with limited incorporation of social markers such as race, gender, and territory. Only 21 studies (17%) evaluated public policies, and 26 (21%) addressed indicators, revealing a scarcity of systematized data to assess policies across different contexts.

Conclusion: The biological approach to disability predominated. Policy evaluation studies identified relevant gaps and barriers in the fulfillment of human rights. Weaknesses were evidenced in data production and in the formulation of indicators across all public policy areas. The findings reinforced the need for more robust, intersectional monitoring models to advance rights and improve inclusive policies.

## Resumen

Objetivo: Mapear la evidencia científica sobre los derechos sociales, las políticas públicas y los indicadores sociales dirigidos a las personas con discapacidad en Brasil, identificando brechas de conocimiento, tendencias metodológicas y oportunidades para mejorar las políticas públicas inclusivas.

Métodos: Se incluyeron estudios publicados desde 2007 hasta abril de 2025, sin restricción de idioma ni de estado de publicación, que abarcaban artículos científicos, disertaciones, tesis, estudios primarios y secundarios, análisis teóricos y documentales, estudios de caso, evaluaciones de políticas, informes y anuarios estadísticos. La selección y la extracción de datos fueron realizadas por cuatro revisores independientes, utilizando la plataforma Rayyan y, posteriormente, hojas de cálculo de Excel. Se incluyeron estudios sobre derechos, políticas públicas e indicadores relacionados con las personas con discapacidad, en diferentes escalas territoriales. Los datos se presentaron de forma narrativa.

Resultados: De los 1.392 estudios identificados, 124 fueron incluidos. Aunque se menciona el modelo social de la discapacidad, predominó el enfoque centrado en la “naturaleza de la discapacidad”, con escasa incorporación de marcadores sociales como la raza, el género y el territorio. Solo 21 estudios (17%) evaluaron políticas públicas y 26 (21%) abordaron indicadores, lo que evidencia una escasez de datos sistematizados para evaluar políticas en distintos contextos.

Conclusión: Predominó el enfoque biológico de la discapacidad. Los estudios de evaluación de políticas identificaron brechas y barreras relevantes en el ejercicio de los derechos humanos. Se evidenciaron debilidades en la generación de datos y en la formulación de indicadores en todas las áreas de políticas públicas. Los hallazgos refuerzan la necesidad de modelos de monitoreo más robustos e interseccionales, contribuyendo a la efectivización de los derechos y al fortalecimiento de políticas inclusivas.

## Introduction

The Convention on the Rights of Persons with Disabilities (CRPD), approved by the United Nations in 2006 and enacted in Brazil as a constitutional amendment [1], is a historical milestone in the global recognition of the human rights of persons with disabilities [2,3]. It marks the paradigmatic transition from the traditional medical model, which characterizes disability exclusively in terms of the individual’s body, to the biopsychosocial approach, which centers on the physical, social, and behavioral barriers that hinder full participation in society with equal rights. This approach also recognizes specificities such as race/color, gender, class, among others, engaging with the theoretical intersectional perspective, which has increasingly been incorporated into disability studies [4-7].

The adoption of the biopsychosocial model has had a profound impact on the design of public policies, as it shifts the focus from curing or restoring the individual’s body toward mitigating environmental barriers. This new paradigm requires that the ways disability is measured and the criteria established for access to public policies take into account the barriers individuals face, rather than being restricted to impairments in body structures and functions.

The Continuous Cash Benefit (*Benefício de Prestação Cotinuada*, BPC) of Social Assistance, which is part of the Basic Social Protection (*Proteção Social Básica*, PSB) of the Unified Social Assistance System (*Sistema Único da Assistência Social*, SUAS), was the first policy to incorporate, in 2009, the biopsychosocial model to assess eligibility, representing an unprecedented advance in the history of social protection and public policy for persons with disabilities in Brazil [8]. Although the country has been a signatory to the CRPD since 2007, disputes over the conceptions guiding access to public policies persist today. The delay in implementing the Unified Biopsychosocial Disability Assessment, which has been under development for several years, is one reflection of this dispute.

The change represented by the biopsychosocial model in the conception of disability and its implications for public policies is substantial. Nevertheless, discrimination, invisibility, and inequality persist [8,9]. Examples include the differences in poverty and social exclusion affecting persons with some disability identified in 2022. Among individuals older than 25 years, 63% had no formal education or had not completed primary education, compared with 32% among persons without disabilities [10].

The more severe the disabilities, the more pronounced the exclusion. The mean real income from the main job among persons older than 14 years with complete or severe disabilities was 45% lower than that of persons without disabilities, a difference that reached 199% when comparing Black women with disabilities to White men without disabilities [11].

The implementation and monitoring of policies aimed at overcoming barriers gain particular relevance in ensuring rights. However, an important practical and scientific gap is the lack of consolidated data and well-defined indicators to support monitoring of public policies for this population group [7,12], which constitutes what may be termed informational invisibility. Data on persons with disabilities have been poorly systematized and, until recently, scattered across several databases, which hindered their use for public policy planning and social oversight.

The National Information System on Disabilities (*Sistema Nacional de Indicadores sobre Deficiência*, Sisdef) emerged as a strategic response [13], in line with the country’s commitments under the CRPD [1] and, in the global context, with the United Nations Sustainable Development Goals [14].

Sisdef is a platform designed to collect, organize, and provide data on public policies for persons with disabilities. Organized into thematic panels on health, education, work, social assistance, demography, and human rights, it includes indicators derived from various public and non-public data sources. Although there are limitations, including partial coverage, outdated data, and gaps in specific areas such as housing, urban mobility, and digital accessibility, its establishment represents a significant advance in addressing the invisibility of persons with disabilities, especially within the framework of the biopsychosocial model.

To support the monitoring of public policies, Sisdef adopted a hierarchical organizational model whose logical framework structures indicators into four major components: situational factors, inputs, processes, and outcomes [15]. The scoping review presented in this article aimed to support improvements to this model.

The objective of this review was to map the scientific evidence on social rights, public policies, and social indicators aimed at persons with disabilities in Brazil, identifying knowledge gaps, methodological trends, and opportunities to improve inclusive public policies.

## Methods

### Study design

It is a scoping review, guided by the methodology of the Joanna Briggs Institute [16] and reported in accordance with the Preferred Reporting Items for Systematic Reviews and Meta-Analyses extension for Scoping Reviews (PRISMA-ScR) checklist [17].

### Research question

This review seeks to answer the question: “What is the scientific production, including grey literature, on rights and public policies related to persons with disabilities after the publication of the CRPD in 2007?”.

This question was constructed based on the PCC acronym, which guides scoping reviews, according to the Joanna Briggs Institute methodology [16].

- Population (P): persons with disabilities.
- Concept (C): scientific production on public policies and rights.
- Context (C): Convention on the Rights of Persons with Disabilities, enacted in December 2006 and published in 2007.

The secondary questions were: What are the theoretical and conceptual approaches/models of disability? Which public policies are addressed in the literature? How are public policies involving persons with disabilities being evaluated? Which indicators have been adopted to assess public policies related to persons with disabilities?

A person with a disability was considered someone who has a long-term impairment of physical, mental, intellectual, or sensory type, which, in interaction with one or more barriers, may hinder their full and effective participation in society on an equal basis with others [1].

### Eligibility criteria

To ensure broad coverage of scientific and technical production on the topic and to respect the linguistic diversity of countries that are signatories to the CRPD, studies published between 2007 and April 2025 in Portuguese, English, Spanish, and French were included. Scientific articles, master’s theses, doctoral dissertations, primary and secondary studies with any methodological approach, theoretical studies, document analyses, case studies, policy evaluations, research reports, and statistical yearbooks were considered. Undergraduate/specialization academic works, editorials, interviews, books, campaign materials, participatory policy documents, preprint articles, and clinical studies or those using the term “disability” in a decontextualized manner were excluded.

### Sources of information

Searches were conducted in the following databases: Medline (via PubMed), Virtual Health Library, Scopus, Web of Science, and Oasisbr (for grey literature). No manual search of references of relevant publications was performed to identify additional potentially eligible studies. No restrictions were applied regarding publication status.

The strategy was developed with the support of a librarian from the National School of Public Health Sérgio Arouca (*Escola Nacional de Saúde Pública Sérgio Arouca*, ENSP) in April 2025. No contact was made with the authors to obtain additional data.

### Search strategies

Controlled descriptors-MeSH (Medical Subject Headings) and DeCS (Health Sciences Descriptors)-were used, combined with free terms and articulated using Boolean operators (AND, OR), and adapted to the specificities of each database. The strategy was designed to ensure comprehensiveness and precision in retrieving relevant studies. The full version of the strategy is available in Supplementary Table 1.

The following limits were applied: language (Portuguese, English, Spanish, and French) and publication period from 2007 to April 2025.

### Selection of evidence sources

This selection was conducted in two stages: (i) title and abstract screening; and (ii) full-text review. Both stages were conducted by pairs of independent reviewers (DMFA and SOP, MFFVSB and MG), using the Rayyan platform. Disagreements were resolved by consensus or by a third reviewer (CMRD). Calibration of criteria was performed using the first 100 studies during screening and 10 studies during eligibility assessment.

Data extraction process

Four researchers (DMFA and SOP, MFFVSB, and MG) independently extracted data from the studies using a standardized, pretested Excel spreadsheet. The spreadsheet was reviewed by a fifth researcher who did not participate in the initial extraction (CMRD). When necessary, this researcher supplemented or adjusted information on the extraction sheet to ensure data standardization. Missing information was recorded as “not reported” or “not applicable.”

### Data items

The following data were extracted.

- General characteristics: authors, title, year, country, CRPD signatory status, cross-country comparisons, objectives, study type, methodological approach, main results, limitations, and recommendations.
- Disability-related characteristics: study population, such as persons with disabilities, their family members, public managers, or other involved groups. Concepts and social markers present in the studies (such as race/ethnicity, gender, disability, health, class/income), as well as situational factors influencing the contexts analyzed.
- Policy characteristics: aspects related to the policies mentioned in the studies, such as level of coverage (from community to global), policy thematic area (health, education, labor, legal, among others), policy timing (whether before or after the CRPD), and policy indicators categorized according to the Sisdef matrix [15].

### Critical appraisal of evidence sources

No methodological quality assessment of the studies was conducted, in accordance with Joanna Briggs Institute guidance for scoping reviews [16].

### Synthesis of results

Scientific production on rights and public policies for persons with disabilities was characterized by theoretical approaches, social markers, and the policies addressed.

To answer the remaining research questions, two subsets were analyzed.

- Policy evaluation studies were described by year, relationship to the CRPD, study type and method, target population, thematic area, and level of coverage.
- Studies addressing indicators related to policies, programs, and actions were systematized by theme and area and classified according to the Sisdef matrix (situational factors, inputs, processes, and outcomes), highlighting gaps in scientific production.

Findings were presented narratively, without meta-analysis, and discussed collectively by the authors. 

### Results 

Of the 1,392 records identified, 864 were selected for title and abstract screening, 207 for full-text assessment, and 124 were included in the review (Figure 1). Among the 124 studies included in the review, 91 were published in peer-reviewed journals. The remaining studies constituted grey literature, comprising 28 master’s theses and 5 doctoral dissertations. Seventy-six studies were published in English, 44 in Portuguese, 3 in French, and 1 in Spanish. The highest number of publications was observed in 2023, 2024, and 2020, with 18, 17, and 13 studies, respectively (Table 1).

**Figure 1:**
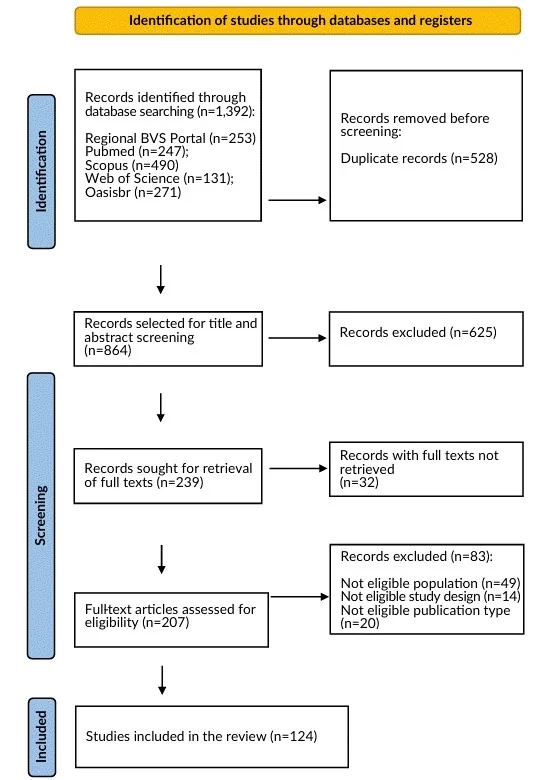
Study selection and inclusion process in the scoping review

**Table 1:** General characteristics of the studies included in the review (n=124)

Author, year (reference)	Country(ies) of the study	Study type/design	Indicators presented?
Mefano, 2014 [7]	Brazil	Policy evaluation	No
Evans et al., 2022 [12]	Australia	Policy evaluation	No
Abreu, 2019 [20]	Brazil	Theoretical discussion	No
Arantes, 2017 [21]	Brazil	Theoretical discussion	No
Arstein-Kerslake et al., 2023 [22]	British Commonwealth of Nations (Commonwealth)	Theoretical discussion	No
Assaf, 2017 [23]	Brazil	Theoretical discussion	No
Breau, 2024 [24]	England	Policy implementation	No
Brehmer-Rinderer, Zigrovic and Weber, 2013 [25]	Spain and Hungary	Policy implementation	No
Colver et al., 2010 [26]	Not identified	Policy implementation	No
Costa, Anache and Sebastián-Heredero, 2023 [27]	Brazil	Theoretical discussion	No
Costa Filho, 2016 [28]	Brazil	Policy implementation	No
Davidson et al., 2016 [29]	Ontario, Canada; Victoria, Australia; England and Wales, United Kingdom; and Northern Ireland	Policy implementation	No
Deb, 2017 [30]	India	Theoretical discussion	No
Díaz et al., 2023 [31]	Spain	Policy implementation	No
Didi, Dowse and Smith, 2018 [32]	Australia	Theoretical discussion	No
Duarte et al., 2017 [33]	Brazil	Theoretical discussion	No
Espín-Tello and Colver, 2017 [34]	France, Ireland, Sweden, England, Northern Ireland, Denmark, Italy and Germany	Not identified	No
Fernandes, 2012 [35]	Brazil	Policy evaluation	No
Ferreira da Mata, 2018 [36]	Portugal	Other (exploratory case study)	No
Fiala, 2019 [37]	Portugal	Policy implementation	No
Fisher et al., 2015 [38]	Australia	Policy implementation	No
Gibson and Mykitiuk, 2012 [39]	Canada	Other (survey and discussion of experiences regarding access to health services)	No
Gill et al., 2020 [40]	Australia	Policy implementation	Yes
Gill et al., 2021 [41]	Australia	Other (case study)	No
Gjertsen, Hardonk and Ineland, 2021 [42]	Iceland, Norway and Sweden	Other (documentary analysis)	No
Gómez et al., 2020 [43]	Not identified	Other (systematic review)	No
Gomes, 2016 [44]	Brazil	Other (case study)	No
Gough and Morris, 2012 [45]	Canada	Other (legislative analysis and key informant access)	No
Grigaitė, 2017 [46]	Lithuania	Policy evaluation	No
Henriques, 2023 [47]	Portugal	Theoretical discussion	No
Hodgson, 2012 [48]	Australia	Theoretical discussion	No
Hoffman, Sritharan and Tejpar, 2016 [49]	Canada	Other (case study)	No
Jaffrès, 2021 [50]	Sweden	Other (case study)	No
Jonasdottir et al., 2021 [51]	Iceland	Theoretical discussion	No
Kelly, 2023 [52]	India	Not identified	No
Mahdanian et al., 2023 [53]	Not identified	Other (review)	No
McCusker et al., 2023 [54]	Czech Republic, England, Finland, Northern Ireland, Norway, Scotland and Slovenia	Other (scoping review preceded by a group discussion with representatives from seven countries)	No
Martins, 2018 [55]	Portugal	Theoretical discussion	No
Matsui, 2013 [56]	Japan	Policy implementation	No
Miranda, 2024 [57]	Portugal	Theoretical discussion	No
Assumpção Lima Momm, Milléo Baract e Godinho Delgado, 2024 [58]	Brazil	Theoretical discussion	No
Munoz-Baell et al., 2008 [59]	Spain	Not identified	Yes
Ngwena, 2013 [60]	South African	Theoretical discussion	No
Oliveira, 2016 [61]	Brazil	Theoretical discussion	No
Pagaime, 2022 [62]	Brazil	Policy implementation	No
Parmenter, 2024 [63]	Global	Theoretical discussion	No
Pérez-Curiel et al., 2023 [64]	Not identified	Other (systematic review)	No
Pinto, 2020 [65]	Morocco and Tunisia	Theoretical discussion	Yes
Saigal and Narayan, 2014 [66]	India	Not identified	No
Sak, 2020 [67]	Brazil	Theoretical discussion	Yes
Samper and Fernández, 2017 [68]	Spain	Theoretical discussion	Yes
Santana, 2016 [69]	Brazil	Other (case study)	Yes
Santiago, 2016 [70]	Portugal	Not identified	No
Santos, 2024 [71]	Cape Verde	Policy evaluation	No
Sequeira, 2015 [72]	Brazil	Other (case study)	No
Silva, 2016 [73]	Brazil	Theoretical discussion	Yes
Silva, 2023 [74]	Brazil	Other (comparative study of legal instruments and pedagogical instruments)	Yes
Silveira, 2017 [75]	Brazil	Theoretical discussion	No
Skempes et al., 2018 [76]	Switzerland	Other (group concept mapping)	Yes
Souza, 2020 [77]	Brazil	Theoretical discussion	No
Srisuppaphon et al., 2017 [78]	Thailand	Policy implementation	No
Symeonidou, 2015 [79]	Cyprus	Other (case study)	
Tibyriçá, 2019 [80]	Brazil	Theoretical discussion	No
Tongsiri et al., 2017 [81]	Thailand	Theoretical discussion	Yes
Trani et al., 2022 [82]	Afghanistan	Theoretical discussion	Yes
Trigueiro, 2014 [83]	Brazil	Not identified	No
von Granitz et al., 2017 [84]	Sweden	Policy evaluation	Yes
Vulcano, 2018 [85]	Brazil	Theoretical discussion	No
Waltz et al., 2018 [86]	Netherlands	Theoretical discussion	No
Winkler et al., 2020 [87]	Czech Republic	Policy evaluation	No
Xavier, 2022 [88]	Brazil	Theoretical discussion	No
Yilmaz, 2020 [89]	Turkey	Policy evaluation	No
García-Piña, 2025 [90]	Mexico	Not identified	No
Gould et al., 2015 [91]	74 countries, grouped into continental regions (Africa, Asia, Europe, Oceania, Latin America and the Caribbean, North America)	Policy implementation	Yes
Magnusson and Bickenbach, 2020 [92]	Sierra Leone	Other (cross-sectional study)	No
Silva, 2023 [93]	Portugal	Policy evaluation	Yes
Wahyudi, Fathan and Kasiyati, 2023 [94]	Indonesia	Policy implementation	No
Wolbring, 2024 [95]	Canada	Other (scoping review)	No
Benedetti and Horvath Júnior, 2024 [96]	Brazil	Policy implementation	Yes
Goodley and Runswick-Cole, 2011 [97]	England	Not identified	No
Njelesani et al., 2024 [98]	Malawi	Theoretical discussion	No
Shikako et al., 2023 [99]	Canada	Policy evaluation	No
Furgalska and Londras, 2024 [100]	England and Wales	Theoretical discussion	No
Lee et al., 2015 [101]	Philippines	Other (case study)	No
Rade et al., 2023 [102]	Ethiopia	Other (cross-sectional study)	No
Coe, 2012 [103]	Armenia, Ethiopia, India, Sierra Leone, and Senegal	Theoretical discussion	No
Eyraud, 2023 [104]	France	Policy evaluation	No
Colon-Cabrera et al., 2021 [105]	Australia	Theoretical discussion	No
Grangaard et al., 2024 [106]	Norway, Sweden, Denmark	Policy implementation	No
Barrera Juarez, 2020 [107]	Brazil	Theoretical discussion	No
Bickenbach, 2011 [108]	Not identified	Policy implementation	Yes
Ferri and Favalli, 2018 [109]	Countries of the European Union	Policy evaluation	No
Ferri and Giannoumis, 2014 [110]	Countries of the European Union	Policy evaluation	No
Garg, Skempes and Bickenbach, 2020 [111]	Sweden, Italy, Germany, Netherlands, and United Kingdom	Policy implementation	Yes
Lord, Stein and Fiala-Butora, 2014 [112]	Not identified	Theoretical discussion	No
Marinho, 2019 [113]	Brazil	Other (integrative review)	No
Maxwell, 2025 [114]	Ireland	Policy evaluation	No
McCarthy, 2019 [115]	Australia	Policy implementation	No
Mira-Aladrén et al., 2024 [116]	Not identified	Policy evaluation	No
Morgan, 2023 [117]	United Arab Emirates	Theoretical discussion	No
Queiroz, 2023 [118]	Brazil	Theoretical discussion	No
Ramos, 2015 [119]	Brazil	Theoretical discussion	No
Resende, 2016 [120]	Brazil	Theoretical discussion	No
Amorim, 2019 [121]	Brazil	Theoretical discussion	Yes
Andrade, 2019 [122]	Brazil	Theoretical discussion	Yes
Cabral, 2022 [123]	Brazil	Theoretical discussion	No
Chagas, 2020 [124]	Brazil	Theoretical discussion	No
Cisternas and Aguila, 2020 [125]	Chile	Policy implementation	No
Curitiba, 2020 [126]	Brazil	Theoretical discussion	No
Durham et al., 2016 [127]	Lao People's Democratic Republic	Theoretical discussion	Yes
Garrido-Cumbrera, Chacón-García and Schädler, 2016 [128]	Spain	Policy implementation	No
Morgan, 2021 [129]	United Arab Emirates	Other (case study)	No
Murphy et al., 2022 [130]	Ireland	Policy implementation	No
Revillard, 2018 [131]	France	Theoretical discussion	No
Elekanachi et al., 2023 [132]	Nigeria	Policy evaluation	No
Grigaitė et al., 2025 [133]	Lithuania	Policy evaluation	Yes
Van Hout et al., 2023 [134]	Malawi	Theoretical discussion	No
Shikako et al., 2023 [135]	Australia, Canada, Fiji, France, Guinea, Haiti, India, Ireland, Jamaica, Malawi, Philippines, Rwanda, South Africa, and Zimbabwe	Policy evaluation	Yes
Stavert et al., 2024 [136]	Scotland	Theoretical discussion	No
Yang, 2024 [137]	Not identified	Theoretical discussion	No
Scherer et al., 2024 [138]	Zambia	Other (scoping review)	No
Schidel, 2023 [139]	Not identified	Theoretical discussion	No
Suleiman et al., 2024 [140]	Global	Policy evaluation	No
Morales, 2022 [141]	Colombia	Policy implementation	No

The included studies were analyzed in relation to theoretical-conceptual approaches, social markers, and public policies addressed. Among the 124 studies, two subsets were defined for specific analyses, in accordance with the research questions: 21 (17%) explicitly addressed public policy evaluation, and 26 (21%) addressed indicators related to policies, programs, and actions targeting persons with disabilities. Seven policy evaluation studies addressed indicators.

The theoretical-conceptual approaches or models of disability were not explicitly stated in the 124 studies assessed, although these studies included analyses of social markers. “Characteristics of disability” was the most frequently addressed marker (56.5%). It was noted that 2.4% mentioned race/color/ethnicity (Table 2).

Two public policies accounted for 50% of the total production: social inclusion and social protection, and inclusive education. The least addressed were those related to access to food and communication policy within the health sector for persons with disabilities (Table 2).

Of the 124 studies included, 21 (16.9%) explicitly reported being policy evaluation studies and were published after 2012, with some increase over the last three years (Table 1).

The majority of the 21 studies used qualitative methods [12,35,46,87,89,93,99,104,109,110,114,116,132,133,135], 3 used mixed methods [7,71,140], and 3 used quantitative methods [81,82,84]. Seven presented indicators [81,82,84,93,99,133,135].

Most studies used primary sources [35,81,82, 84,87,89,93,99,104,109,114,133]. Seven were theoretical in type [7,12,35,99,109,110,140]; 6 included public policy managers, policymakers, and implementers/operators [46,81,82,84,87,89]. One study included, in addition to managers, persons with disabilities [46]. Eight studies, produced after 2023, included persons with disabilities [71,93,104,114,116,132,133,135].

In terms of policy thematic areas, social protection/ inclusion [46,71,82,84,104,116,132,133] and health [7,12,87,99,114,135] were most prominent. There was 1 institutional study conducted in Lithuania, in which two independent living homes were examined [46]. The remaining studies had national or continental scope.

**Table 2:** Distribution of studies according to identified social markers and public policies addressed (n=124)

Social markers and policies addressed^a^	n (%)
**Social markers**	
Characteristics of disability^b^	70 (56.5)
Age group/generation^b^	14 (11.3)
Sex/gender^b^	13 (10.5)
Class/income/social status^b^	7 (5.6)
Health conditions^b^	7 (5.6)
Nationality/territoriality^b^	7 (5.6)
Race/color/ethnicity^b^	3 (2.4)
Other conditions of vulnerability^b^	6 (5.0)
No marker addressed^b^	10 (8.1)
Markers not identified^b^	
**Policies addressed**	
Social inclusion and protection policy^c^	25 (20.1)
Inclusive education policy^c^	36 (29.0)
Health policy^c^	26 (21.0)
Work and employment inclusion policy^c^	24 (19.4)
Accessibility policy^c^	22 (17.7)
Implementation and monitoring of the Convention on the Rights of Persons with Disabilities^c^	6 (4.8)
Food access policy	9 (7.3)
Health communication policy^c^	1 (0.8)
Other policies^c^	1 (0.8)
Policies not identified	10 (8.0)

^a^Studies may have been included in more than one category.

^b^Social markers used to characterize disability.

^c^Policy areas addressed.

All studies adopted human and social rights as an analytical framework. Eleven emphasized gaps in the policies studied [7,12,35,46,71,84,87,99,116,135,140].

Four highlighted potentialities or improvements in the evaluated policy field [82,109,110,140].

Of the 124 studies included, 26 (21%) addressed indicators related to policies, programs, and actions for persons with disabilities. The characterization of these studies, including the topic addressed, the information provided, the indicators presented, and the policy thematic area, may be requested from the authors.

Definitions, methods, and topics were heterogeneous. Often, a single study fell under more than one policy thematic area. Among the 26 studies with indicators, most addressed social protection/inclusion [30,31,65, 82,84,91,93,96,121,127,133]. Two studies [76,108]

proposed indicator lists, one based on the International Classification of Functioning, Disability and Health and another focused on rehabilitation.

The 26 studies were mapped and organized by thematic areas into four major categories and items adapted from the logical framework of the Sisdef model [8] to identify gaps (Table 3).

No studies were identified in 8 of the Sisdef matrix subitems: 1 in situational factors (professional teams); 1 in inputs (intersectoral and/or interinstitutional partnerships); 4 in process (infrastructure expansion, accessibility improvements, acquisition of accessible vehicles, and expansion of teams); and 2 in outcomes (adequacy of collective and public-use spaces).

**Table 3:** Distribution of studies addressing indicators, according to thematic area and categories of the logical framework of the National Information System on Disabilities (Sisdef) (n=26)

Matrix^a^	Social protection and inclusionn (%)	Education	Health	Work	Legal	Housing	CRPD monitoring^c^
	n (%)	n (%)	n (%)	n (%)	n (%)	n (%)	n (%)
**Situational factors**							
1. Policy implementation	7 (27)	5 (19)	5 (19)	5 (19)	2 (8)	1 (4)	1 (4)
2. Accessibility conditions	3 (12)	1 (4)	0 (0)	1 (4)	0 (0)	0 (0)	0 (0)
3. Professional teams	0 (0)	0 (0)	0 (0)	0 (0)	0 (0)	0 (0)	0 (0)
4. Institutional/legal definitions	1 (4)	4 (15)	2 (8)	2 (8)	0 (0)	0 (0)	0 (0)
5. Social needs	0 (0)	0 (0)	0 (0)	0 (0)	0 (0)	0 (0)	0 (0)
6. Public data	1 (4)	2 (8)	1 (4)	3 (12)	0 (0)	0 (0)	0 (0)
**Inputs**							
0. Not applicable^b^	3 (12)	2 (8)	1 (4)	3 (12)	0 (0)	0 (0)	0 (0)
1. Infrastructure	2 (8)	1 (4)	1 (4)	0 (0)	0 (0)	1 (4)	0 (0)
2. Human resources	0 (0)	0 (0)	0 (0)	1 (4)	1 (4)	0 (0)	0 (0)
3. Intersectoral and/or interinstitutional partnerships	0 (0)	0 (0)	0 (0)	0 (0)	0 (0)	0 (0)	0 (0)
4. Standards and/or protocols	6 (23)	4 (15)	5 (19)	4 (15)	1 (4)	0 (0)	1 (4)
5. Information system	2 (8)	1 (4)	1 (4)	1 (4)	0 (0)	0 (0)	0 (0)
**Processes**							
0. Not applicable^b^	6 (23)	3 (12)	6 (23)	4 (15)	2 (8)	0 (0)	0 (0)
1. Infrastructure expansion	0 (0)	0 (0)	0 (0)	0 (0)	0 (0)	0 (0)	0 (0)
2. Accessibility improvements	0 (0)	0 (0)	0 (0)	0 (0)	0 (0)	0 (0)	0 (0)
3. Team for communicational and informational accessibility	0 (0)	1 (4)	0 (0)	0 (0)	0 (0)	0 (0)	0 (0)
4. Intersectoral and interinstitutional partnerships for infrastructure works	0 (0)	0 (0)	0 (0)	0 (0)	0 (0)	1 (4)	0 (0)
5. Acquisition of affordable vehicles	0 (0)	0 (0)	0 (0)	0 (0)	0 (0)	0 (0)	0 (0)
6. Expansion of teams	0 (0)	0 (0)	0 (0)	0 (0)	0 (0)	0 (0)	0 (0)
7. Continuing education	2 (8)	1 (4)	0 (0)	0 (0)	1 (4)	0 (0)	0 (0)
8. Addressing stigma/discrimination	4 (15)	2 (8)	1 (4)	4 (15)	0 (0)	0 (0)	0 (0)
**Results**							
0. Not applicable^b^	1 (4)	0 (0)	3 (12)	0 (0)	0 (0)	0 (0)	0 (0)
1. Territorial distribution	2 (8)	2 (8)	1 (4)	1 (4)	0 (0)	0 (0)	0 (0)
2. Expansion of access and quality	2 (8)	2 (8)	2 (8)	1 (4)	0 (0)	0 (0)	0 (0)
3. Adequacy of collective and public spaces	0 (0)	0 (0)	0 (0)	0 (0)	0 (0)	0 (0)	0 (0)
4. Expansion of services	1 (4)	1 (4)	1 (4)	1 (4)	0 (0)	0 (0)	0 (0)
5. Strengthening of networks	1 (4)	2 (8)	2 (8)	2 (8)	0 (0)	0 (0)	0 (0)
6. Reduction of complaints	1 (4)	1 (4)	1 (4)	1 (4)	0 (0)	0 (0)	0 (0)
7. Advances in the CRPD^c^ and LBI^d^	8 (31)	4 (15)	3 (12)	7 (27)	2 (8)	1 (4)	1 (4)

^a^Studies may have been included in more than one category.

^b^When indicators did not fit into any related category of the Sisdef framework.

^c^Convention on the Rights of Persons with Disabilities.

^d^Brazilian Inclusion Law.

### Discussion

This review, which sought to map scientific production on rights and public policies for persons with disabilities after the CRPD, found that the defense of the rights of persons with disabilities and the adoption of the social model of disability as a theoretical perspective were present in the studies. However, the intersection of disability with other social markers was not frequent, which demonstrated a strong predominance of the “characteristics of disability” as the sole marker.

The evaluation of public policies for persons with disabilities was present in only a fraction of the studies, and the use of policy-related indicators was scarce. In analogy with the Sisdef matrix, gaps were identified across situational factors, inputs, processes, and outcomes. Qualitative studies predominated, and the most frequent thematic areas were inclusive education and social protection and inclusion.

A key limitation is the risk of missing grey literature and the language restriction.

Although the studies included in the review were aligned with the social model of disability, a biomedical approach was predominant, even though it is recognized that social markers such as race/color, gender, and class impact access to services and rights guaranteed by law. Policy analyses have not incorporated disability from an intersectional perspective, which considers the lived experience of the intersection of different social markers in everyday life and in population groups with trajectories of oppression [4-7].

Among studies reporting evaluation as an objective or study design (21 studies), there was considerable diversity in themes and methodological choices. Evaluation is a practice present across multiple domains, which may generate conceptual and methodological polysemy in policy evaluation, requiring explicit clarification of approaches [142].

Among the 21 evaluation studies, non-population-based studies predominated. Among population-based studies, 8-the majority-included persons with disabilities, 6 involved managers, policymakers, and policy implementers, and 1 involved both groups of actors. Considering, according to the Public Policies Pentagon [143], that public policies involve actions and interventions planned and carried out by the State or governments, as well as interactions among the actors involved in policies, it is relevant to note that persons with disabilities, as recipients of the policies studied, and actors-individual or collective-“endowed with resources (...), a certain degree of autonomy, strategies, and capacity to make choices” were the most frequently present in evaluations.

A predominance of studies focusing on national-level policy evaluation was observed. Considering the CRPD as the main framework of this review, which constitutes a commitment among State Parties, the results highlighted the delimitation of objects and levels of evaluation as a way to frame the reality under study [142]. Scientific production on rights and public policies related to persons with disabilities after the Convention involves a multiplicity of policy thematic areas and an inclusive perspective as a guiding principle or desired goal. Only a few studies were found in the policy area addressing justice.

Evaluation studies, by pointing to gaps and relevant barriers in the realization of human rights ideals and definitions for persons with disabilities, as well as indicating advances and good practices, have revealed that the Convention has served as a benchmark for policy evaluation. It established a new human rights lexicon that moves beyond the deficit-based perspective on disability and advances the consolidation of policy models anchored in the principles of equality and nondiscrimination [1].

One of the main findings of the review identified that, although there has been growing scientific production on the rights of persons with disabilities since the adoption of the Convention, there are still significant gaps in the use of indicators to monitor and evaluate these policies. Only 26 of the 124 included studies directly addressed the use of indicators, and only 7 of the 21 policy evaluation studies addressed them explicitly.

The review also highlighted the absence of standards for measuring and comparing progress and setbacks in public policies across regions and levels of government. This was mentioned in documents that highlighted difficulties in monitoring progress in implementing the Convention using a set of common indicators [136], as well as in specific areas, such as indicators for intellectual disability [140] and mental health [134]. The lack of standardized indicators hindered accurate assessment of the impacts of these policies on the lives of persons with disabilities, as well as comparisons across different contexts and levels of data aggregation.

Some proposals are being developed to support this standardization, such as the WHO Model Disability Survey for identifying persons with disabilities, which includes the degree of disability and actions to ensure equal rights in society, based on the International Classification of Functioning, Disability and Health [137], and the World Health Organization’s disability and functioning disaggregation tool [87].

Two studies proposed standardized lists of indicators: one based on the International Classification of Functioning, Disability and Health [108], and the other focused on rehabilitation, presenting a framework of indicators developed by a group of experts [76].

Most other studies analyzed data from various sources, including country documents (laws and implemented policies), disability-related statistics, censuses, information systems, interviews, and data from specific programs for persons with disabilities at the local and national levels, making comparative analyses across datasets infeasible.

The lack of information and indicators related to persons with disabilities-that is, informational invisibility-is an important finding of this study, as although the Convention was published 17 years ago, most signatory countries still do not have sufficiently robust population data to evaluate and monitor policies. This delay may negatively impact people’s lives. A study that analyzed public policies in 14 countries during the COVID-19 pandemic and compared them with States’ obligations under the Convention observed that, even with public health policies implemented for the general population, it is necessary to expand these policies to address the needs of persons with disabilities [142].

The cross-analysis of indicators aligned with the dimensions of the Sisdef matrix reinforced the perception of existing gaps in policy evaluation for persons with disabilities across the proposed categories (situational factors, inputs, processes, and outcomes). The findings pointed to key weaknesses that must be addressed to improve data sources and the production of population indicators that support decision-making in public policies.

Research on indicators for monitoring and evaluation remains relatively underexplored, given the volume of publications. Similarly, policy evaluation studies were not predominant. There are no standardized tools to assess and compare progress or setbacks in implemented policies or actions, which hampers the monitoring of CRPD implementation progress using a common set of indicators.

Mapping the scientific production related to rights and public policies for persons with disabilities after the CRPD made it possible to reveal the main policy themes of interest in the academic literature, as well as the still incipient adoption and standardization of indicators for policy analysis. This topic deserves attention on the scientific research agenda to advance perspectives on the rights of persons with disabilities and on social indicators for evaluating public policies targeting this population.

**Ethical aspects**

This research used publicly available and anonymized databases.

**Protocol registration**

The protocol was registered on the Zenodo platform under registration number 7789108 (https://zenodo.org/search?q=7789108&l=list&p=1&s=10&sort=bestmatch).

**Use of generative artificial intelligence**

Not used.

## Data Availability

Data will be made available upon request and subject to the authors’ evaluation at the following e-mail address: nippis@fiocruz.br.
