# Supplementary material for: Social rights, public policies, and social indicators related to persons with disabilities in Brazil: a scoping review
Source: Epidemiol Serv Saude. 2026 Jul 22;35:e20250679. doi: 10.1590/S2237-96222026v35e20250679.en (PMC13686386; doi:10.1590/S2237-96222026v35e20250679.en)
Supplement: Translation

# Translation: Direitos sociais, políticas públicas e indicadores sociais relacionados a pessoas com deficiência no Brasil: revisão de escopo

**Authors:** Cristina Maria Rabelais Duarte, Dolores Maria Franco de Abreu, Mariana Gabriel, Silvia de Oliveira Pereira, Maria Fernanda de Faria von Sydow Bittencourt, Daniele da Silva Garcez Novaes, Maritsa Carla de Bortoli

**Affiliations:** 1 Fundação Oswaldo Cruz, Núcleo de Informação, Política Pública e Inclusão Social, Rio de Janeiro, RJ, Brazil. Fundação Oswaldo Cruz Fundação Oswaldo Cruz Núcleo de Informação, Política Pública e Inclusão Social Rio de Janeiro RJ Brazil; 2 Fundação Oswaldo Cruz, Núcleo de Informação, Política Pública e Inclusão Social, Rio de Janeiro, RJ, Brazil. Fundação Oswaldo Cruz Fundação Oswaldo Cruz Núcleo de Informação, Política Pública e Inclusão Social Rio de Janeiro RJ Brazil; 3 Fundação Oswaldo Cruz, Núcleo de Informação, Política Pública e Inclusão Social, Rio de Janeiro, RJ, Brazil. Fundação Oswaldo Cruz Fundação Oswaldo Cruz Núcleo de Informação, Política Pública e Inclusão Social Rio de Janeiro RJ Brazil; 4 Fundação Oswaldo Cruz, Núcleo de Informação, Política Pública e Inclusão Social, Rio de Janeiro, RJ, Brazil. Fundação Oswaldo Cruz Fundação Oswaldo Cruz Núcleo de Informação, Política Pública e Inclusão Social Rio de Janeiro RJ Brazil; 5 Centro Universitário UNIFASE, Núcleo de Informação, Política Pública e Inclusão Social, Rio de Janeiro, RJ, Brazil. Centro Universitário UNIFASE Núcleo de Informação, Política Pública e Inclusão Social Rio de Janeiro RJ Brazil; 6 Fundação Oswaldo Cruz, Núcleo de Informação, Política Pública e Inclusão Social, Rio de Janeiro, RJ, Brazil. Fundação Oswaldo Cruz Fundação Oswaldo Cruz Núcleo de Informação, Política Pública e Inclusão Social Rio de Janeiro RJ Brazil; 7 Fundação Oswaldo Cruz, Núcleo de Informação, Política Pública e Inclusão Social, Rio de Janeiro, RJ, Brazil. Fundação Oswaldo Cruz Fundação Oswaldo Cruz Núcleo de Informação, Política Pública e Inclusão Social Rio de Janeiro RJ Brazil

**Keywords:** Pessoa com Deficiência, Política Pública, Indicadores Sociais, Revisão de Escopo, Inclusão Social.

## Abstract

Objetivo: Mapear as evidências científicas sobre direitos sociais, políticas públicas e indicadores sociais voltados às pessoas com deficiência no Brasil, identificando lacunas de conhecimento, tendências metodológicas e oportunidades para aprimorar as políticas públicas inclusivas.

Métodos: Foram incluídos estudos publicados a partir de 2007 até abril de 2025, sem restrição de idioma ou status de publicação, abrangendo artigos científicos, dissertações, teses, estudos primários e secundários, análises teóricas e documentais, estudos de caso, avaliações de políticas, relatórios e anuários estatísticos. A seleção e a extração dos dados foram realizadas por quatro revisores independentes, utilizando a plataforma Rayyan e, posteriormente, planilhas Excel. Foram incluídos estudos sobre direitos, políticas públicas e indicadores relacionados a pessoas com deficiência, em diferentes escalas territoriais. Os dados foram apresentados de forma narrativa.

Resultados: Dos 1.392 estudos identificados, 124 foram incluídos. Embora o modelo social da deficiência seja citado, predominou a abordagem centrada na “natureza da deficiência”, com baixa incorporação de marcadores sociais como raça, gênero e território. Apenas 21 estudos (17%) avaliaram políticas públicas e 26 (21%) abordaram indicadores, revelando escassez de dados sistematizados para avaliar políticas em diferentes contextos.

Conclusão: Predominou a abordagem biológica da deficiência. Os estudos de avaliação de políticas apontaram lacunas e barreiras relevantes no cumprimento de direitos humanos. Evidenciaram-se fragilidades na produção de dados e na formulação de indicadores em todas as áreas de políticas públicas. Os achados reforçaram a necessidade de modelos de monitoramento mais robustos e interseccionais, o que contribui para efetivar direitos e aprimorar políticas inclusivas.

## Introdução

A Convenção sobre os Direitos das Pessoas com Deficiência (CDPD), aprovada pela Organização das Nações Unidas em 2006 e promulgada no Brasil com status de emenda constitucional [1], é um marco his-tórico no reconhecimento global dos direitos humanos das pessoas com deficiência [2,3]. Ela marca a tran-sição paradigmática do modelo médico tradicional, que caracteriza deficiência exclusivamente a partir do corpo do indivíduo, para a abordagem biopsicossocial, que confere centralidade às barreiras físicas, sociais e atitudinais as quais são obstáculos à plena participação na sociedade com igualdade de direitos. Essa aborda-gem também reconhece especificidades como raça/cor da pele, gênero, classe, entre outras, dialogando com a perspectiva teórica da interseccionalidade, que vem sendo incorporada nos estudos sobre deficiência [4-7].

A adoção do modelo biopsicossocial tem reflexo profundo no desenho de políticas públicas, pois desloca o foco da cura ou da recuperação do corpo do indivíduo para mitigar barreiras no ambiente. Esse novo paradig-ma requer que as formas de mensurar a deficiência e o estabelecimento de critérios para acesso a políticas públicas considerem as barreiras enfrentadas pelas pessoas, em vez de se restringirem a alterações em estruturas e funções do corpo.

O Benefício de Prestação Continuada da Assistência Social, que integra a Proteção Social Básica do Sistema Único de Assistência Social, foi a primeira política a incorporar, em 2009, o modelo biopsicossocial para avaliar a elegibilidade, o que representou um avanço sem precedentes na história da proteção social e da política pública voltada para pessoas com deficiência no Brasil [8]. Entretanto, apesar de o país ser signatário da CDPD desde 2007, prevalece ainda hoje uma disputa em torno das concepções que orientam o acesso a políticas públicas. A demora em se instituir a Avaliação Biopsicossocial Unificada da Deficiência, em construção há alguns anos, é um dos reflexos dessa disputa.

É expressiva a mudança representada pelo modelo biopsicossocial na concepção de deficiência e seus reflexos sobre políticas públicas. Entretanto, discrimina-ção, invisibilidade e desigualdade ainda persistem [8,9]. Tomem-se como exemplos as diferenças na situação de pobreza e exclusão social que marcavam pessoas com algum tipo de deficiência identificadas em 2022. Entre os maiores de 25 anos, 63% não tinham instrução ou ensino fundamental completo, em comparação a 32% entre as pessoas sem deficiência [10].

Quanto mais graves forem as deficiências, mais notá-vel é a exclusão. O rendimento médio real do trabalho principal de pessoas com deficiências completas ou graves com mais de 14 anos era 45% menor do que o de pessoas sem deficiência, diferença que chegava a 199% ao se comparar mulheres pretas com deficiência com homens brancos sem deficiência [11].

A implementação e o monitoramento de políticas para superar barreiras ganham especial relevância, para garantir direitos. Contudo, uma lacuna prática e científica importante é a ausência de dados consolidados e indica-dores bem-definidos que sustentem o monitoramento de políticas públicas voltadas para o segmento [7,12], configurando o que se pode denominar de invisibilidade informacional. Dados sobre pessoas com deficiência são pouco sistematizados e, até recentemente, dispersos em diversas bases, o que dificultou a sua utilização para o planejamento de políticas públicas e o controle social.

O Sistema Nacional de Indicadores sobre Deficiência (Sisdef) surge como resposta estratégica [13], em con-sonância com os compromissos assumidos pelo país na CDPD [1] e, no contexto global, com os objetivos do desenvolvimento sustentável da Organização das Nações Unidas [14].

O Sisdef é uma plataforma para coletar, organizar e disponibilizar dados sobre políticas públicas para pessoas com deficiência. Organizado em painéis temáticos nas áreas de saúde, educação, trabalho, assistência social, de-mografia e direitos humanos, traz indicadores construídos a partir de diferentes fontes de dados, públicas ou não. Embora haja limitações como cobertura parcial e desatualização dos dados, e lacunas em determinadas áreas, como habitação, mobilidade urbana e acessibilidade digital, sua constituição representa expressivo avanço para enfrentar a invisibilidade de pessoas com deficiência, especialmente sob o respaldo do modelo biopsicossocial.

Buscando favorecer o monitoramento de políticas públicas, o Sisdef adotou um modelo de organização hierárquica, cuja matriz lógica organiza os indicadores em grandes componentes: fatores situacionais, insumos, processos e resultados [15]. A revisão de escopo apresentada neste artigo teve a finalidade de subsidiar o aprimoramento desse modelo.

O objetivo desta revisão foi mapear as evidências científicas sobre direitos sociais, políticas públicas e indicadores sociais voltados às pessoas com deficiên-cia no Brasil, identificando lacunas de conhecimento, tendências metodológicas e oportunidades para apri-moramento das políticas públicas inclusivas.

## Métodos

### Delineamento

Trata-se de revisão de escopo, norteada pela meto-dologia do Joanna Briggs Institute [16] e relatada de acordo com o checklist do Preferred Reporting Items for Systematic Reviews and Meta-Analyses for Scoping Reviews (PRISMA-ScR) [17].

### Pergunta de pesquisa

Esta revisão busca responder à pergunta: “Qual é a produção científica, incluindo literatura cinzenta, sobre direitos e políticas públicas relacionadas à pessoa com deficiência após a publicação da CDPD, em 2007?”.

Essa pergunta foi construída com base no acrôni-mo PCC, que orienta revisões de escopo, conforme a metodologia do Joanna Briggs Institute [16].

- População (P): pessoas com deficiência.
- Conceito (C): produção científica sobre políticas públicas e direitos.
- Contexto (C): Convenção sobre os Direitos das Pessoas com Deficiência, promulgada em de-zembro de 2006 e publicada em 2007.

As perguntas secundárias foram: Quais as aborda-gens/modelos teórico-conceituais da deficiência? Quais políticas públicas são consideradas nas produções? Como políticas públicas que envolvem pessoas com deficiência estão sendo avaliadas? Quais indicadores têm sido adotados para avaliar políticas públicas rela-cionadas às pessoas com deficiência?

Considerou-se pessoa com deficiência aquela que tem impedimento de longo prazo de natureza física, mental, intelectual ou sensorial, o qual, em interação com uma ou mais barreiras, pode obstruir sua partici-pação plena e efetiva na sociedade em igualdade de condições com as demais pessoas [1].

### Critérios de elegibilidade

Buscando garantir ampla cobertura da produção científica e técnica sobre o tema e respeitando a di-versidade linguística dos países signatários da CDPD, foram incluídos estudos publicados entre 2007 e abril de 2025, nos idiomas português, inglês, espanhol e francês. Foram considerados artigos científicos, dissertações de mestrado, teses de doutorado, estudos primários e secundários com qualquer abordagem metodológica, estudos teóricos, análises documentais, estudos de caso, avaliações de políticas, relatórios de pesquisa e anuá-rios estatísticos. Foram excluídos trabalhos acadêmicos de graduação/especialização, editoriais, entrevistas, livros, materiais de campanhas, documentos político-participativos, artigos em *preprint* e estudos clínicos ou com uso não contextualizado do termo “deficiência”.

### Fontes de informação

As buscas foram realizadas nas bases de dados Medline (via PubMed), Biblioteca Virtual em Saúde, Scopus, Web of Science e Oasisbr (para literatura cin-zenta). Não foi realizada busca manual nas referências de publicações relevantes para identificar estudos adi-cionais potencialmente elegíveis. Não foram aplicadas restrições ao status de publicação.

A estratégia foi elaborada com apoio de bibliotecário da Escola Nacional de Saúde Pública Sérgio Arouca em abril de 2025. Não houve contato com autores para obtenção de dados adicionais.

### Estratégias de busca

Utilizaram-se descritores controlados - MeSH (Medical Subject Headings) e DeCS (Descritores em Ciências da Saúde) -, combinados a termos livres, articu-lados por operadores booleanos (AND, OR) e adaptados às especificidades de cada base de dados. A formulação da estratégia buscou garantir abrangência e precisão na recuperação dos estudos relevantes. A versão completa da estratégia está disponível na Tabela Suplementar 1.

Foram aplicados os seguintes limites: idioma (português, inglês, espanhol e francês) e período de publicação compreendido entre 2007 e abril de 2025.

### Seleção das fontes de evidência

Essa seleção foi realizada em duas etapas: (i) tria-gem de títulos e resumos; e (ii) leitura do texto completo. Ambas as etapas foram conduzidas por duplas de revisoras independentes (DMFA e SOP, MFFVSB e MG), utilizando a plataforma Rayyan. Divergências foram resolvidas por consenso ou por terceira revisora (CMRD). A calibração dos critérios foi feita com os 100 primeiros estudos na triagem e 10 estudos na elegibilidade.

### Processo de extração dos dados

Quatro pesquisadoras (DMFA e SOP, MFFVSB e MG) extraíram os dados dos estudos de forma in-dependente, utilizando planilha Excel padronizada e previamente testada. A planilha foi revisada por uma quinta pesquisadora que não participou da extração inicial (CMRD). Quando necessário, esse pesquisador complementou ou ajustou informações na planilha de extração, assegurando a padronização dos dados coletados. Informações ausentes foram registradas como “não reportado” ou “não se aplica”.

### Itens de dados

Foram extraídos os dados a seguir.

- Características gerais: autores, título, ano, país, signatário da CDPD, comparações entre países, objetivos, tipo de estudo, abordagem metodológica, principais resultados, limitações e recomendações apresentadas.
- Características relacionadas à deficiência: popu-lação estudada, como pessoas com deficiência, seus familiares, gestores públicos ou outros grupos envolvidos. Conceitos e marcadores sociais que atravessam os estudos (como raça/ etnia, gênero, deficiência, saúde, classe/renda), além dos fatores situacionais que influenciam os contextos analisados.
- Características das políticas públicas: aspectos ligados às políticas mencionadas nos estudos, como o nível de abrangência (desde o comunitário até o global), a área temática da política (saúde, educação, trabalho, jurídica, entre outras), a tem-poralidade da política (se anterior ou posterior à CDPD) e os indicadores de política, categorizados segundo a matriz do Sisdef [15].

### Avaliação crítica das fontes de evidência

Não foi realizada avaliação da qualidade metodo-lógica dos estudos, conforme orientação do Instituto Joanna Briggs para revisões de escopo [16].

### Síntese dos resultados

A produção científica sobre direitos e políticas públicas para pessoas com deficiência foi caracterizada pelas abordagens teóricas, marcadores sociais e polí-ticas abordadas.

Para responder às demais perguntas de pesquisa, dois subconjuntos foram analisados.

Os estudos de avaliação de políticas foram descritos segundo ano, relação com a CDPD, tipo e método de estudo, população-alvo, área temática e nível de abrangência.

Os estudos que abordavam indicadores rela-cionados a políticas, programas e ações foram sistematizados por tema e área e classificados conforme a matriz do Sisdef (fatores situacionais, insumos, processos e resultados), evidenciando lacunas na produção científica.

Os achados foram apresentados de forma narrativa, sem meta-análise, e discutidos coletivamente pelos autores.

## Resultados

Dos 1.392 registros encontrados, 864 foram sele-cionados para avaliação de título e resumo, 207 para avaliação do texto completo e 124 foram incluídos na revisão (Figura 1). Entre os 124 estudos incluídos na revisão, 91 foram publicados em periódicos. Os de-mais eram literatura cinzenta, sendo 28 dissertações e 5 teses. Setenta e seis foram publicados em inglês, 44 em português, 3 em francês e 1 em espanhol. Observou-se a maior quantidade de publicação em 2023, 2024 e 2020, com 18, 17 e 13 estudos (Tabela 1).

**Figura 1:** Processo de seleção e inclusão dos estudos na revisão de escopo

**Tabela 1:** Características gerais dos estudos incluídos na revisão (n=124)

Autor, ano (referência)	País(es) do estudo	Tipo de estudo/desenho	Apresenta indicadores?
Mefano, 2014 [7]	Brasil	Avaliação de políticas	Não
Evans et al., 2022 [12]	Austrália	Avaliação de políticas	Não
Abreu, 2019 [20]	Brasil	Discussão teórica	Não
Arantes, 2017 [21]	Brasil	Discussão teórica	Não
Arstein-Kerslake et al., 2023 [22]	Comunidade Britânica de Nações (Commonwealth)	Discussão teórica	Não
Assaf, 2017 [23]	Brasil	Discussão teórica	Não
Breau, 2024 [24]	Inglaterra	Implementação de políticas	Não
Brehmer-Rinderer, Zigrovic e Weber, 2013 [25]	Espanha e Hungria	Implementação de políticas	Não
Colver et al., 2010 [26]	Não identificado	Implementação de políticas	Não
Costa, Anache e Sebastián-Heredero, 2023 [27]	Brasil	Discussão teórica	Não
Costa Filho, 2016 [28]	Brasil	Implementação de políticas	Não
Davidson et al., 2016 [29]	Ontário, Canadá; Victoria, Austrália; Inglaterra e País de Gales, Reino Unido; e Irlanda do Norte	Implementação de políticas	Não
Deb, 2017 [30]	Índia	Discussão teórica	Não
Díaz et al., 2023 [31]	Espanha	Implementação de políticas	Não
Didi, Dowse e Smith, 2018 [32]	Austrália	Discussão teórica	Não
Duarte et al., 2017 [33]	Brasil	Discussão teórica	Não
Espín-Tello e Colver, 2017 [34]	França, Irlanda, Suécia, Inglaterra, Irlanda do Norte, Dinamarca, Itália e Alemanha	Não identificado	Não
Fernandes, 2012 [35]	Brasil	Avaliação de políticas	Não
Ferreira da Mata, 2018 [36]	Portugal	Outro (estudo de caso exploratório)	Não
Fiala, 2019 [37]	Portugal	Implementação de políticas	Não
Fisher et al., 2015 [38]	Austrália	Implementação de políticas	Não
Gibson e Mykitiuk, 2012 [39]	Canadá	Outro (levantamento e discussão de experiências sobre acesso a serviços de saúde)	Não
Gill et al., 2020 [40]	Austrália	Implementação de políticas	Sim
Gill et al., 2021 [41]	Austrália	Outro (estudo de caso)	Não
Gjertsen, Hardonk e Ineland, 2021 [42]	Islândia, Noruega e Suécia	Outro (estudo documental)	Não
Gómez et al., 2020 [43]	Não identificado	Outro (revisão sistemática)	Não
Gomes, 2016 [44]	Brasil	Outro (estudo de caso)	Não
Gough e Morris, 2012 [45]	Canadá	Outro (análise legislativa e acesso a informantes-chave)	Não
Grigaitė, 2017 [46]	Lituânia	Avaliação de políticas	Não
Henriques, 2023 [47]	Portugal	Discussão teórica	Não
Hodgson, 2012 [48]	Austrália	Discussão teórica	Não
Hoffman, Sritharan e Tejpar, 2016 [49]	Canadá	Outro (estudo de caso)	Não
Jaffrès, 2021 [50]	Suécia	Outro (estudo de caso)	Não
Jonasdottir et al., 2021 [51]	Islândia	Discussão teórica	Não
Kelly, 2023 [52]	Índia	Não identificado	Não
Mahdanian et al., 2023 [53]	Não identificado	Outro (revisão)	Não
McCusker et al., 2023 [54]	República Tcheca, Inglaterra, Finlândia, Irlanda do Norte, Noruega, Escócia e Eslovênia	Outro (revisão de escopo antecedida de debate em grupo com representantes de 7 países)	Não
Martins, 2018 [55]	Portugal	Discussão teórica	Não
Matsui, 2013 [56]	Japão	Implementação de políticas	Não
Miranda, 2024 [57]	Portugal	Discussão teórica	Não
Assumpção Lima Momm, Milléo Baract e Godinho Delgado, 2024 [58]	Brasil	Discussão teórica	Não
Munoz-Baell et al., 2008 [59]	Espanha	Não identificado	Sim
Ngwena, 2013 [60]	África do Sul	Discussão teórica	Não
Oliveira, 2016 [61]	Brasil	Discussão teórica	Não
Pagaime, 2022 [62]	Brasil	Implementação de políticas	Não
Parmenter, 2024 [63]	Global	Discussão teórica	Não
Pérez-Curiel et al., 2023 [64]	Não identificado	Outro (revisão sistemática)	Não
Pinto, 2020 [65]	Marrocos e Tunísia	Discussão teórica	Sim
Saigal e Narayan, 2014 [66]	Índia	Não identificado	Não
Sak, 2020 [67]	Brasil	Discussão teórica	Sim
Samper e Fernández, 2017 [68]	Espanha	Discussão teórica	Sim
Santana, 2016 [69]	Brasil	Outro (estudo de caso)	Sim
Santiago, 2016 [70]	Portugal	Não identificado	Não
Santos, 2024 [71]	Cabo Verde	Avaliação de políticas	Não
Sequeira, 2015 [72]	Brasil	Outro (estudo de caso)	Não
Silva, 2016 [73]	Brasil	Discussão teórica	Sim
Silva, 2023 [74]	Brasil	Outro (estudo comparativo de instrumentos legais e instrumentos pedagógicos)	Sim
Silveira, 2017 [75]	Brasil	Discussão teórica	Não
Skempes et al., 2018 [76]	Suíça	Outro (mapeamento de conceito de grupo)	Sim
Souza, 2020 [77]	Brasil	Discussão teórica	Não
Srisuppaphon et al., 2017 [78]	Tailândia	Implementação de políticas	Não
Symeonidou, 2015 [79]	Chipre	Outro (estudo de caso)	
Tibyriçá, 2019 [80]	Brasil	Discussão teórica	Não
Tongsiri et al., 2017 [81]	Tailândia	Discussão teórica	Sim
Trani et al., 2022 [82]	Afeganistão	Discussão teórica	Sim
Trigueiro, 2014 [83]	Brasil	Não identificado	Não
von Granitz et al., 2017 [84]	Suécia	Avaliação de políticas	Sim
Vulcano, 2018 [85]	Brasil	Discussão teórica	Não
Waltz et al., 2018 [86]	Holanda	Discussão teórica	Não
Winkler et al., 2020 [87]	República Tcheca	Avaliação de políticas	Não
Xavier, 2022 [88]	Brasil	Discussão teórica	Não
Yilmaz, 2020 [89]	Turquia	Avaliação de políticas	Não
García-Piña, 2025 [90]	México	Não identificado	Não
Gould et al., 2015 [91]	74 países, agrupados em regiões continentais (África, Ásia, Europa, Oceania, América Latina e Caribe, América do Norte)	Implementação de políticas	Sim
Magnusson e Bickenbach, 2020 [92]	Serra Leoa	Outro (estudo transversal)	Não
Silva, 2023 [93]	Portugal	Avaliação de políticas	Sim
Wahyudi, Fathan e Kasiyati, 2023 [94]	Indonésia	Implementação de políticas	Não
Wolbring, 2024 [95]	Canadá	Outro (revisão de escopo)	Não
Benedetti e Horvath Júnior, 2024 [96]	Brasil	Implementação de políticas	Sim
Goodley e Runswick-Cole, 2011 [97]	Inglaterra	Não identificado	Não
Njelesani et al., 2024 [98]	Maláui	Discussão teórica	Não
Shikako et al., 2023 [99]	Canadá	Avaliação de políticas	Não
Furgalska e Londras, 2024 [100]	Inglaterra e País de Gales	Discussão teórica	Não
Lee et al., 2015 [101]	Filipinas	Outro (estudo de caso)	Não
Rade et al., 2023 [102]	Etiópia	Outro (transversal)	Não
Coe, 2012 [103]	Armênia, Etiópia, Índia, Serra Leoa e Senegal	Discussão teórica	Não
Eyraud, 2023 [104]	França	Avaliação de políticas	Não
Colon-Cabrera et al., 2021 [105]	Austrália	Discussão teórica	Não
Grangaard et al., 2024 [106]	Noruega, Suécia, Dinamarca	Implementação de políticas	Não
Barrera Juarez, 2020 [107]	Brasil	Discussão teórica	Não
Bickenbach, 2011 [108]	Não identificado	Implementação de políticas	Sim
Ferri e Favalli, 2018 [109]	Países da União Europeia	Avaliação de políticas	Não
Ferri e Giannoumis, 2014 [110]	Países da União Europeia	Avaliação de políticas	Não
Garg, Skempes e Bickenbach, 2020 [111]	Suécia, Itália, Alemanha, Países Baixos e Reino Unido	Implementação de políticas	Sim
Lord, Stein e Fiala-Butora, 2014 [112]	Não identificado	Discussão teórica	Não
Marinho, 2019 [113]	Brasil	Outro (revisão integrativa)	Não
Maxwell, 2025 [114]	Irlanda	Avaliação de políticas	Não
McCarthy, 2019 [115]	Austrália	Implementação de políticas	Não
Mira-Aladrén et al., 2024 [116]	Não identificado	Avaliação de políticas	Não
Morgan, 2023 [117]	Emirados Árabes	Discussão teórica	Não
Queiroz, 2023 [118]	Brasil	Discussão teórica	Não
Ramos, 2015 [119]	Brasil	Discussão teórica	Não
Resende, 2016 [120]	Brasil	Discussão teórica	Não
Amorim, 2019 [121]	Brasil	Discussão teórica	Sim
Andrade, 2019 [122]	Brasil	Discussão teórica	Sim
Cabral, 2022 [123]	Brasil	Discussão teórica	Não
Chagas, 2020 [124]	Brasil	Discussão teórica	Não
Cisternas e Aguila, 2020 [125]	Chile	Implementação de políticas	Não
Curitiba, 2020 [126]	Brasil	Discussão teórica	Não
Durham et al., 2016 [127]	República Democrática Popular do Laos	Discussão teórica	Sim
Garrido-Cumbrera, Chacón-García e Schädler, 2016 [128]	Espanha	Implementação de políticas	Não
Morgan, 2021 [129]	Emirados Árabes	Outro (estudo de caso)	Não
Murphy et al., 2022 [130]	Irlanda	Implementação de políticas	Não
Revillard, 2018 [131]	França	Discussão teórica	Não
Elekanachi et al., 2023 [132]	Nigéria	Avaliação de políticas	Não
Grigaitė et al., 2025 [133]	Lituânia	Avaliação de políticas	Sim
Van Hout et al., 2023 [134]	Maláui	Discussão teórica	Não
Shikako et al., 2023 [135]	Austrália, Canadá, Ilhas Fiji, França, Guiné, Haiti, Índia, Irlanda, Jamaica, Maláui, Filipinas, Ruanda, África do Sul e Zimbábue	Avaliação de políticas	Sim
Stavert et al., 2024 [136]	Escócia	Discussão teórica	Não
Yang, 2024 [137]	Não identificado	Discussão teórica	Não
Scherer et al., 2024 [138]	Zâmbia	Outro (revisão de escopo)	Não
Schidel, 2023 [139]	Não identificado	Discussão teórica	Não
Suleiman et al., 2024 [140]	Global	Avaliação de políticas	Não
Morales, 2022 [141]	Colômbia	Implementação de políticas	Não

Os estudos incluídos foram analisados em relação às abordagens teórico-conceituais, marcadores sociais e políticas públicas abordadas. Entre os 124, foram definidos dois subconjuntos para análises específicas, considerando as perguntas de investigação: 21 (17%) que tratavam explicitamente da avaliação de políticas públicas e 26 (21%) que abordavam indicadores rela-cionados a políticas, programas e ações voltadas às pessoas com deficiência. Sete estudos sobre avaliação de políticas abordavam indicadores.

As abordagens ou os modelos teórico-conceituais de deficiência não foram explicitados nos 124 artigos avaliados, embora trouxessem análises relacionadas a marcadores sociais. A “natureza da deficiência” foi o marcador mais abordado (56,5%). Destacou-se que 2,4% citaram “raça/cor da pele/etnia” (Tabela 2).

Duas políticas públicas corresponderam a 50% do total das produções: inclusão e proteção social e educação inclusiva. As menos abordadas foram aquelas relacionadas ao acesso à alimentação e à política de comunicação no âmbito da saúde para a pessoa com deficiência (Tabela 2).

Dos 124 estudos incluídos, 21 (16,9%) informaram explicitamente serem de avaliação de políticas e foram publicados após 2012, com algum crescimento nos últimos três anos (Tabela 1).

A maioria dos 21 estudos utilizou métodos qualitativos [12,35,46,87,89,93,99,104, 109,110,114,116,132,133,135], 3 utilizaram métodos

mistos [7,71,140] e 3 métodos quantitativos [81,82,84]. Sete apresentaram indicadores [81,82,84,93,99,^133^,^135^].

Parte majoritária dos estudos utilizou fontes pri-márias [35,81,82,84,87,89,93,99,104,109,114,133].

Sete foram de cunho teórico [7,12,35,99, 109,110,140]; 6 incluíram gestores, formuladores e implementadores/ operadores de políticas públicas [46,81,82,84,87,89]. Um incluiu, além de gestores, pessoas com deficiência [46]. Oito, produzidos após 2023, incluíram pessoas com deficiência [71,93,104,114,116,132,133,135].

Destacaram-se, como áreas temáticas de políticas, a proteção/inclusão social [46,71, 82,84,104,116,132,133] e a saúde [7,12,87,99,114,135]. Houve 1 estudo institucional, realizado na Lituânia, onde duas Casas de Vida Independente foram estudadas [46]. O restante foi de abrangência nacional ou continental.

**Tabela 2:** Distribuição dos estudos, segundo marcadores sociais identificados e políticas públicas abordadas (n=124)

Marcadores sociais e políticas abordadas^a^	n (%)
Marcadores sociais	
Natureza da deficiência^b^	70 (56,5)
Faixa etária/geração^b^	14 (11,3)
Sexo/gênero^b^	13 (10,5)
Classe/renda/posição social^b^	7 (5,6)
Condições de saúde^b^	7 (5,6)
Nacionalidade/territorialidade^b^	7 (5,6)
Raça/cor da pele/etnia^b^	3 (2,4)
Outras condições de vulnerabilidade^b^	6 (5,0)
Não abordaram marcador^b^	10 (8,1)
Marcadores não identificados^b^	25 (20,1)
Políticas abordadas	
Política de inclusão e proteção socialc	36 (29,0)
Política de educação inclusivac	26 (21,0)
Política de saúdec	24 (19,4)
Política de inclusão no trabalho e emprego^c^	22 (17,7)
Política de acessibilidade^c^	6 (4,8)
Implementação e monitoramento da Convenção dos Direitos da Pessoa com Deficiência^c^	9 (7,3)
Política de acesso à alimentação	1 (0,8)
Política de comunicação no âmbito da saúde^c^	1 (0,8)
Outras políticas^c^	10 (8,0)

^a^Os estudos podem ter sido incluídos em mais de uma categoria; ^b^Marcadores sociais utilizados para caracterizar a deficiência; ^c^Áreas de políticas abordadas.

Todos os estudos adotaram direitos humanos e sociais como parâmetro analítico. Onze enfatizaram lacunas nas políticas estudadas [7,12,35,46,71,84,87,99,116,135,140]. Quatro enfatizam potencialida-des ou melhorias no campo das políticas avaliadas [82,109,110,140].

Dos 124 estudos incluídos, 26 (21%) abordaram indicadores relacionados a políticas, programas e ações para pessoas com deficiência. A caracterização desses estudos, segundo assunto abordado, lista de informa-ções e indicadores apresentados e área temática de política, pode ser solicitada aos autores.

As definições, os métodos e os temas foram heterogêneos. Muitas vezes um estudo entrou em mais de uma área temática de política. Dos 26 estu-dos com indicadores, a maioria abordou proteção/ inclusão social [30,31,65,82,84,91,93,96,121,127,133]. Dois [76,108] propuseram listas de indicado-res, um com base na Classificação Internacional de Funcionalidade, Incapacidade e Saúde e outro sobre a temática da reabilitação.

Os 26 estudos foram sistematizados por áreas temáticas e organizados segundo quatro grandes categorias e itens adaptados da matriz lógica do modelo Sisdef [8], com a finalidade de identificar lacunas (Tabela 3).

Não foram identificados estudos em 8 dos subi-tens da matriz do Sisdef: 1 em fatores situacionais (equipes profissionais); 1 em insumos (parcerias intersetoriais e/ou interinstitucionais); 4 em pro-cesso (ampliação de infraestrutura, melhorias de acessibilidade, aquisição de veículos acessíveis e ampliação de equipes); e 2 em resultados (adequação de espaços coletivos e de uso público).

**Tabela 3:** Distribuição dos estudos que abordam indicadores, segundo área temática e categorias da matriz lógica do Sistema Nacional de Indicadores sobre Deficiência (Sisdef) (n=26)

Matriza	Proteção e inclusão social	Educação	Saúde	Trabalho	Jurídicos	Habitação	Monitoramento da CDPDc
	n (%)	n (%)	n (%)	n (%)	n (%)	n (%)	n (%)
Fatores situacionais							
1. Implementação de políticas	7 (27)	5 (19)	5 (19)	5 (19)	2(8)	1 (4)	1 (4)
2. Condições de acessibilidade	3 (12)	1 (4)	0 (0)	1 (4)	0 (0)	0 (0)	0 (0)
3. Equipes profissionais	0 (0)	0 (0)	0 (0)	0 (0)	0 (0)	0 (0)	0 (0)
4. Definições institucionais/legais	1 (4)	4 (15)	2 (8)	2 (8)	0 (0)	0 (0)	0 (0)
5. Necessidades sociais	0 (0)	0 (0)	0 (0)	0 (0)	0 (0)	0 (0)	0 (0)
6. Dados públicos	1 (4)	2 (8)	1 (4)	3 (12)	0 (0)	0 (0)	0 (0)
Insumos							
0. Não se aplica^b^	3 (12)	2 (8)	1 (4)	3 (12)	0 (0)	0 (0)	0 (0)
1. Infraestrutura	2 (8)	1 (4)	1 (4)	0 (0)	0 (0)	1 (4)	0 (0)
2. Recursos humanos	0 (0)	0 (0)	0 (0)	1 (4)	1 (4)	0 (0)	0 (0)
3. Parcerias intersetoriais e/ou interinstitucionais	0 (0)	0 (0)	0 (0)	0 (0)	0 (0)	0 (0)	0 (0)
4. Normas e/ou protocolos	6 (23)	4 (15)	5 (19)	4 (15)	1 (4)	0 (0)	1 (4)
5. Sistema de informação	2 (8)	1 (4)	1 (4)	1 (4)	0 (0)	0 (0)	0 (0)
Processos							
0. Não se aplica^b^	6 (23)	3 (12)	6 (23)	4 (15)	2 (8)	0 (0)	0 (0)
Ampliação de infraestrutura	0 (0)	0 (0)	0 (0)	0 (0)	0 (0)	0 (0)	0 (0)
Melhorias em acessibilidade	0 (0)	0 (0)	0 (0)	0 (0)	0 (0)	0 (0)	0 (0)
3. Equipe para acessibilidade comunicacional e informacional	0 (0)	1 (4)	0 (0)	0 (0)	0 (0)	0 (0)	0 (0)
4. Parcerias intersetoriais e interinstitucionais para obras	0 (0)	0 (0)	0 (0)	0 (0)	0 (0)	1 (4)	0 (0)
5. Aquisição de veículos acessíveis	0 (0)	0 (0)	0 (0)	0 (0)	0 (0)	0 (0)	0 (0)
6. Ampliação de equipes	0 (0)	0 (0)	0 (0)	0 (0)	0 (0)	0 (0)	0 (0)
7. Educação permanente	2 (8)	1 (4)	0 (0)	0 (0)	1 (4)	0 (0)	0 (0)
8. Enfrentamento ao preconceito	4 (15)	2 (8)	1 (4)	4 (15)	0 (0)	0 (0)	0 (0)
Resultados							
0. Não se aplica ^b^	1 (4)	0 (0)	3 (12)	0 (0)	0 (0)	0 (0)	0 (0)
1. Distribuição territorial	2 (8)	2 (8)	1 (4)	1 (4)	0 (0)	0 (0)	0 (0)
2. Ampliação do acesso e qualidade	2 (8)	2 (8)	2 (8)	1 (4)	0 (0)	0 (0)	0 (0)
3. Adequação de espaços coletivos	0 (0)	0 (0)	0 (0)	0 (0)	0 (0)	0 (0)	0 (0)
4. Ampliação de atendimentos	1 (4)	1 (4)	1 (4)	1 (4)	0 (0)	0 (0)	0 (0)
5. Fortalecimento de redes	1 (4)	2 (8)	2 (8)	2 (8)	0 (0)	0 (0)	0 (0)
6. Redução de denúncias	1 (4)	1 (4)	1 (4)	1 (4)	0 (0)	0 (0)	0 (0)
7. Avanços na CDPD^c^ e LBI^d^	8 (31)	4 (15)	3 (12)	7 (27)	2 (8)	1 (4)	1 (4)

^a^Os estudos podem ter sido incluídos em mais de uma categoria;

^b^Quando os indicadores não se enquadravam em nenhuma categoria relacionada da matriz do Sisdef;

^c^Convenção sobre os Direitos da Pessoa com Deficiência;

^d^Lei Brasileira de Inclusão.

## Discussão 

Esta revisão, que buscou mapear a produção cien-tífica relacionada aos direitos e às políticas públicas para pessoas com deficiência após a CDPD, identificou que a defesa dos direitos das pessoas com deficiên-cia e a adoção do modelo social da deficiência como perspectiva teórica estiveram presentes nos estudos. Entretanto, a intersecção da condição de deficiência com outros marcadores sociais não foi frequente, o que demonstrou forte presença da natureza da deficiência como único marcador.

A avaliação de políticas públicas voltadas para pessoas com deficiência esteve presente em apenas uma fração dos estudos, e a abordagem de indicadores relaciona-dos a políticas mostrou-se escassa. Em analogia com a matriz do Sisdef, verificaram-se lacunas relativas a fatores situacionais, insumos, processos e resultados. Houve predominância de estudos qualitativos, e as áreas temáticas mais frequentes nos estudos foram relativas à educação inclusiva e à proteção e inclusão social.

Vale sublinhar como limitação o risco de perda na seleção da literatura cinzenta, bem como na restrição de idiomas.

Apesar dos estudos da revisão estarem alinhados com o modelo social da deficiência, houve predominância da abordagem biológica, mesmo sendo reconhecido que marcadores sociais, como raça/cor da pele, gê-nero e classe, impactam o acesso a serviços e direitos garantidos por lei. As análises de políticas não têm in-corporado a deficiência em perspectiva interseccional, que considera a vivência da intersecção de diferentes marcadores sociais no cotidiano de pessoas e nos seg-mentos populacionais com trajetória de opressão [4-7].

Nos estudos que informaram adotar a avaliação como objetivo ou desenho de estudo (21 estudos), observou-se a diversidade de temas e opções meto-dológicas. A avaliação é prática existente em diversos âmbitos, o que pode gerar polissemia conceitual e metodológica na área de avaliação de políticas reque-rendo explicitação das abordagens [142].

Entre os 21 estudos de avaliação, houve predomi-nância de estudos não populacionais. Entre os estudos populacionais, 8, que representam a maioria, incluí-ram pessoas com deficiência, 6 envolveram gestores, formuladores e implementadores de políticas, e 1 en-volveu esses dois conjuntos de atores. Considerando, conforme o Pentágono das Políticas Públicas [143], que políticas públicas envolvem ações e intervenções planejadas e realizadas pelo Estado ou governos, como também interações entre os atores envolvidos nas políticas, é relevante observar que pessoas com defi-ciência, como destinatárias das políticas estudadas, e atores, individuais ou coletivos “dotados de recursos (...) certa autonomia, estratégias e capacidade de fazer escolhas” foram os mais presentes nas avaliações.

Observou-se predominância de estudos voltados para a avaliação de políticas de abrangência nacional. Considerando a CDPD como o principal marco desta revisão, constituindo um compromisso entre esta-dos-partes, os resultados destacaram a delimitação dos objetos e dos níveis de avaliação como forma de recorte da realidade que se pretende estudar [142]. A produção científica sobre direitos e política públicas relacionadas à pessoa com deficiência após a publi-cação da Convenção envolve uma multiplicidade de áreas temáticas de políticas públicas e a perspectiva inclusiva como parâmetro ou imagem-objetivo a alcan-çar. Ressalta-se que foram encontrados poucos estudos na área temática da política que aborda a justiça.

Os estudos de avaliação, ao apontarem a existência de lacunas e barreiras relevantes no cumprimento dos ideais e das definições de direitos humanos das pessoas com deficiência, assim como ao indicarem alguns avanços e boas práticas, revelaram que a Convenção constituiu parâmetro para a avalição de políticas. Ela estabeleceu um novo léxico nos direitos humanos, que supera a perspectiva da deficiência como déficit e avança para a consolidação de modelos de políticas ancorados nos princípios de igualdade e não discriminação [1].

Um dos principais resultados da revisão identificou que, embora exista crescente produção científica so-bre os direitos das pessoas com deficiência desde a promulgação da Convenção, ainda há lacunas signifi-cativas no uso de indicadores para monitorar e avaliar essas políticas. Somente 26 dos 124 estudos incluídos abordaram diretamente o uso de indicadores, e apenas 7 dos 21 estudos que avaliavam políticas também o abordaram diretamente.

A revisão também destacou a ausência de padrões para medir e comparar avanços e retrocessos nas políticas públicas em diferentes regiões e esferas governamentais. Isso foi mencionado em documen-tos que apontaram a dificuldade em monitorar o progresso na implementação da Convenção com relação a um conjunto de indicadores comuns [136] e em algumas áreas específicas como indicadores para a deficiência intelectual [140] e a saúde mental [134]. A falta de indicadores padronizados dificultou a avaliação precisa dos impactos dessas políticas na vida das pessoas com deficiência, bem como a comparação entre diferentes contextos e níveis de agregação de dados.

Algumas propostas estão sendo publicadas para colaborar nessa padronização, como o WHO Model Disability Survey na identificação das pessoas com deficiência, que inclui o grau da deficiência e a ação para garantir direitos em condições de igualdade na so-ciedade, tendo como base a Classificação Internacional de Funcionalidade, Incapacidade e Saúde [137] e a ferramenta de desagregação de funcionalidade e de-ficiência da Organização Mundial da Saúde [87].

Dois estudos propuseram listas padronizadas de indi-cadores a serem utilizados: um apontou a Classificação Internacional de Funcionalidade, Incapacidade e Saúde como base [108], e o outro focou a temática da reabilitação, apresentando um quadro de indicadores elaborado por um grupo de especialistas [76].

Os outros estudos, em sua maioria, analisaram di-ferentes dados oriundos de documentos dos países (leis e políticas implementadas), estatísticas relacio-nadas a pessoa com deficiência, censo, sistemas de informações, entrevistas e dados de programas espe-cíficos para pessoas com deficiência em nível local e nacional, impossibilitando realizar análises comparativas entre os dados apresentados.

A falta de informação e indicadores relacionados à pessoa com deficiência, ou seja, a invisibilidade in-formacional, é evidência importante deste estudo, pois, embora a Convenção tenha sido publicada há 17 anos, a maioria dos países signatários não tem dados populacionais de qualidade satisfatória para avaliar e monitorar políticas. Esse atraso pode ter impacto negativo na vida das pessoas. Um estudo, que analisou as políticas públicas de 14 países durante a pandemia da covid-19 e fez um paralelo com as obrigações dos estados no âmbito da Convenção, observou que, mes-mo com as políticas públicas de saúde implementadas para a população como um todo, é necessário ampliar essas políticas para responder às necessidades da vida de pessoas com deficiência [142].

O cruzamento dos indicadores identificados com as dimensões da matriz do Sisdef reforçou a percepção das lacunas existentes no campo da avaliação de políticas públicas para pessoas com deficiência em cada uma das categorias propostas (fatores situacionais, insumos, processos e resultados). Os achados apontaram as prin-cipais fragilidades a serem enfrentadas para aprimorar fontes de dados e a própria produção de indicadores populacionais que auxiliem a tomada de decisão em políticas públicas.

A produção sobre indicadores para o monitoramento e a avaliação revelou-se um campo ainda pouco explo-rado, considerando o volume de artigos. Da mesma forma, os estudos de avaliação de políticas foram pouco predominantes. Quanto aos indicadores, observou-se ausência de utilização de padrões para avaliar e com-parar possíveis avanços e retrocessos das políticas ou ações implementadas, situação que dificulta monitorar o progresso na implementação da Convenção consi-derando um conjunto de indicadores comuns.

O mapeamento da produção científica relaciona-da aos direitos e às políticas públicas para pessoas com deficiência após a CDPD possibilitou revelar as principais temáticas de políticas de interesse na lite-ratura acadêmica, bem como as, ainda incipientes, adoção e padronização de indicadores para análise de políticas. O tema merece destaque na agenda da pesquisa científica, para que novos estudos avancem nas perspectivas sobre os direitos das pessoas com deficiência e de indicadores sociais que possam ser utilizados para avaliar políticas públicas voltadas para essa população.

**Aspectos éticos**

Esta pesquisa utilizou bancos de dados de domínio público e anonimizados.

**Registro do protocolo**

O protocolo foi registrado na plataforma Zenodo, sob o número de registro 7789108 (https://zenodo.org/search?q=7789108&l=list&p=1&s=10&sort=bestmatch).

**Uso de inteligência artificial generativa**

Não empregada.

## References

[1] 1 Brasil. Convenção sobre os direitos das pessoas com deficiência. Lei brasileira de inclusão da pessoa com deficiência (Estatuto da Pessoa com Deficiência). Brasília; 2016 17 ago. 2025. https://www.mds.gov.br/webarquivos/Oficina%20PCF/JUSTI%C3%87A%20E%20CIDADANIA/convencao-e-lbi-pdf.pdf.

[2] 2 DHANDA, A. Construindo um novo léxico dos direitos humanos: Convenção sobre os Direitos das Pessoas com Deficiências. Sur, Rev int direitos human. 2008;5(8):42-59.

[3] 3 DINIZ, D. O que é deficiência. São Paulo: Editora Brasiliense, 2007.

[4] 4 AKOTIRENE, C. Interseccionalidade. São Paulo: Pólen Editorial, 2019.

[5] 5 COLLINS, PH. Interseccionalidade. São Paulo: Boitempo, 2020.

[6] 6 CRENSHAW, K. Documento para o encontro de especialistas em aspectos da discriminação racial relativos ao gênero. Rev Estud Fem. 2002;171-88.

[7] 7 MEFANO, V. Política pública para a pessoa com deficiência: Brasil e experiência internacional. Dissertação (Mestrado) - Escola Nacional de Saúde Pública Sergio Arouca, Rio de Janeiro, 2014. 29 abr. 2025. https://arca.fiocruz.br/items/4ad283b7-6d2d-4c04-9b1c-2e2c61d2027d.

[8] 8 DUARTE, CMR; MARCELINO, MA; BOBBOLINI, CS; BOCCOLINI, PMM. Proteção social e política pública para populações vulneráveis: uma avaliação do Benefício de Prestação Continuada da Assistência Social - BPC no Brasil. Cien Saude Colet. 2017;22(11):3515-26.10.1590/1413-812320172211.2209201729211157

[9] 9 NOGUEIRA, GC; SCHOELLER, SD; RAMOS, FRS; PADILHA, MI; BREHMER, LCF; MARQUES, AMFB. Perfil das pessoas com deficiência física e Políticas Públicas: a distância entre intenções e gestos. Cien Saude Colet. 2016;21(10):3131-42.10.1590/1413-812320152110.1762201627783786

[10] 10 Instituto Brasileiro de Geografia e Estatística. Censo Demográfico 2022: pessoas com deficiência e pessoas diagnosticadas com transtorno do espectro autista - Resultados preliminares da amostra. Brasília: IBGE, 2025 6 nov. 2025. https://biblioteca.ibge.gov.br/index.php/biblioteca-catalogo?view=detalhes&id=2102178.

[11] 11 Instituto Brasileiro de Geografia e Estatística. Pessoas com deficiência 2022: PNAD Contínua: divulgação dos resultados gerais. Rio de Janeiro: IBGE, 2023 6 nov. 2025. https://biblioteca.ibge.gov.br/index.php/biblioteca-catalogo?view=detalhes&id=2102013.

[12] 12 EVANS, E; HOWLETT, S; KREMSTER, T; SIMPSON, J; KAYESS, R; TROLLOR, J. Service development for intellectual disability mental health: a human rights approach. J. Intellect. Dis. Res. 2012;56(11):1098-109.10.1111/j.1365-2788.2012.01636.x23106753

[13] 13 Núcleo de Informação, Políticas Públicas e Inclusão Social. Sobre o Sisdef. Nippis, 2021 10 set. 2024. https://nippis.icict.fiocruz.br/sisdef/.

[14] 14 Organização das Nações Unidas. Transformando nosso mundo: a Agenda 2030 para o desenvolvimento sustentável. Nova Iorque: ONU, 2015 6 nov. 2025. https://brasil.un.org/sites/default/files/2020-09/agenda2030-pt-br.pdf.

[15] 15 DIAS, FS. Proposta de modelo para criação, monitoramento e avaliação de políticas públicas relacionadas às pessoas com deficiência. Petrópolis: Nippis, 2023.

[16] 16 PETERS, M; GODFREY, C; MCINERNEY, P; MUNN, Z; TRICCO, A; KHALIL, H. Scoping reviews: JBI Manual for Evidence Synthesis. JBI Global Wiki, 2020 11 set. 2024. https://synthesismanual.jbi.global. https://doi.org/10.46658/JBIMES-20-12.

[17] 17 TRICCO, AC; LILLIE, E; ZARIN, W; O'BRIEN, KK; COLQUHOUN, H; LEVAC, D; et al. PRISMA Extension for Scoping Reviews (PRISMA-ScR): checklist and explanation. Ann Intern Med. 2018;169(7):467-73.10.7326/M18-085030178033

[18] 18 OUZZANI, M; HAMMADY, H; FEDOROWICZ, Z; ELMAGARMID, A. Rayyan: a web and mobile app for systematic reviews. Systematic Reviews. 2016;5(1):210.10.1186/s13643-016-0384-4PMC513914027919275

[19] 19 PAGE, MJ; MCKENZIE, JE; BOSSUYT, PM; BOUTRON, I; HOFFMANN, TC; MULROW, CD; et al. The PRISMA 2020 statement: an updated guideline for reporting systematic reviews. BMJ. 2021;372(71).10.1136/bmj.n71PMC800592433782057

[20] 20 ABREU, TPC. Acesso das pessoas com deficiência mental aos direitos e garantias previstos na Lei Brasileira de Inclusão por meio do Sistema Único de Assistência Social. Saúde em Debate. 2019;43(n. especial 4):190-206.

[21] 21 ARANTES, ML. A inclusão das pessoas com deficiência na comunidade: o direito à moradia e as barreiras que impedem a sua efetivação [trabalho de conclusão de curso]. Vitória: Faculdade de Direito de Vitória, 2017 29 abr. 2025. http://repositorio.fdv.br:8080/handle/fdv/13/simple-search?query=&sort_by=score&order=desc&rpp=10&filter_field_1=advisor&filter_type_1=equals&filter_value_1=Bussinguer%2C+Elda+Coelho+de+Azevedo&etal=0&filtername=author&filterquery=Arantes%2C+Maristela+Lugon&filtertype=equals.

[22] 22 ARSTEIN-KERSLAKE, A; MAKER, Y; DEUTSCHMANN, A; RICHARDSON, S. Criminalisation of sex with disabled people with cognitive impairments in Commonwealth countries: a colonial remnant that interferes with the human right to sexual agency. Int J Disabil Soc Justice. 2023;3(2):4-25.16.

[23] 23 ASSAF, DL. A escolarização do aluno com síndrome de Down e o ensino especializado. Dissertação (Mestrado) - Universidade Metodista de São Paulo, São Paulo, 2017. 29 abr. 2025. https://repositorio.metodista.br/items/ab5de0ae-3ee3-48a9-9869-6a5fdbfe5fab.

[24] 24 BREAU, G. Health systems, health policies, and health issues for people with intellectual disabilities in England. J Policy Pract Intellect Disabil. 2024;21(1).

[25] 25 BREHMER-RINDERER, B; ZIGROVIC, L; NAUE, U; WEBER, G. Promoting health of persons with intellectual disabilities using the UN convention on the rights of persons with disabilities: Early implementation assessment in Spain and Hungary. J Policy Pract Intellect Disabil. 2013;10(1):25-36.

[26] 26 COLVER, AF; DICKINSON, HO; PARKINSON, K; ARNAUD, C; BECKUNG, E; FAUCONNIER, J; et al. Access of children with cerebral palsy to the physical, social and attitudinal environment they need: a cross-sectional European study. J Disabil Rehabil. 2010;33(1):28-35.10.3109/09638288.2010.48566920446803

[27] 27 COSTA, JA; ANACHE, AA; SEBASTIÁN-HEREDERO, E. Accessibility and justice in higher education for students with visual disabilities: a human rights issue. Rev. Diálogos e Perspectivas. Nov 24 2023;10(2):39-50.

[28] 28 COSTA FILHO, WM. O desafio da educação inclusiva para crianças com deficiência intelectual: um estudo do caso da Escola Estadual Marta da Conceição em Belém-Pará-Amazônia. 2016 29 abr. 2025. https://repositorio.unb.br/bitstream/10482/23203/1/2016_WaldirMacieiradaCostaFilho.pdf.

[29] 29 DAVIDSON, G; BROPHY, L; CAMPBELL, J; FARRELL, SJ; GOODING, P; O'BRIEN, AMAM. An international comparison of legal frameworks for supported and substitute decision-making in mental health services. Int J Law and Psychiatry. 2016;44:30-40.10.1016/j.ijlp.2015.08.02926318975

[30] 30 DEB, S. SDG's indicators framework and disability in India. Indian J. Hum. Dev. 2017;11(2):232-50.

[31] 31 DÍAZ, MP; NOELL, JF; DÍAZ-GAROLERA, G; ROVIRA, CP. Are social services for people with intellectual disabilities aligned with the rights model? Opinions of people, their families and professionals. Alternativas. 2023;30(2):381-408.

[32] 32 DIDI, A; DOWSE, L; SMITH, L. Intellectual disability and complex support needs: human rights perspective for policy and practice. Intl J Hum Rts. 2018;22(8):989-1006.

[33] 33 DUARTE, CMR; MARCELINO, MA; BOCCOLINI, CS; BOCCOLINI, PMM. Proteção social e política pública para populações vulneráveis: uma avaliação do Benefício de Prestação Continuada da Assistência Social - BPC no Brasil TT. Ciênc Saúde Colet (Impr). 2017;22(11):3515-26.10.1590/1413-812320172211.2209201729211157

[34] 34 ESPÍN-TELLO, SM; COLVER, A. How available to European children and young people with cerebral palsy are features of their environment that they need? Res Dev Disabil. 2017;71:1-10.10.1016/j.ridd.2017.09.018PMC567935828987966

[35] 35 FERNANDES, SFP. A formação de professores de ciências biológicas e a educação inclusiva: uma interface da formação inicial e continuada. 2012 29 abr. 2025. https://repositorio.bc.ufg.br/tedeserver/api/core/bitstreams/52775d61-a67a-4d1a-9b5b-1c9da52497ef/content.

[36] 36 MATA, HCCCF. Educação inclusiva e alunos com Currículo Específico Individual (CEI): percepções da comunidade educativa. 2018 29 abr. 2025. https://repositorio.ipv.pt/bitstreams/cf9b27e7-1481-4edd-82d6-d3632ef7604a/download.

[37] 37 FIALA, E. A critical analysis of the implementation of the right to work and employment in the national context of Germany and Portugal. Implications for Social Policy. 2019 29 abr. 2025. https://scholar.google.com/citations?view_op=view_citation&hl=de&user=mtyqm2QAAAAJ&citation_for_view=mtyqm2QAAAAJ:u5HHmVD_uO8C.

[38] 38 FISHER, KR; LUTZ, D; GADOW, F; ROBINSON, S; GENDERA, S. A transformative framework for deinstitutionalisation. Res Pract Intellect Dev Disabil. 2015;2(1):60-72.

[39] 39 GIBSON, BE; MYKITIUK, R. Health care access and support for disabled women in Canada: falling short of the UN Convention on the Rights of Persons with Disabilities: a qualitative study. Women's Health Issues. 2012;22(1):E111-8.10.1016/j.whi.2011.07.01121968028

[40] 40 GILL, NS; AMOS, A; MUHSEN, H; HATTON, J; EKANAYAKE, C; KISELY, S. Measuring the impact of revised mental health legislation on human rights in Queensland, Australia. Int J Law Psychiatry. 2020;73:101634.10.1016/j.ijlp.2020.10163433068843

[41] 41 GILL, NS; PARKER, S; AMOS, A; LAKEMAN, R; EMELEUS, M; BROPHY, L; et al. Opening the doors: critically examining the locked wards policy for public mental health inpatient units in Queensland Australia. Aust. New Zealand J Psychiatr. 2021;55(9):844-8.10.1177/0004867421102561934159793

[42] 42 GJERTSEN, H; HARDONK, S; INELAND, J. Work inclusion for people with intellectual disabilities in three Nordic countries: the current policy and challenges. Scand J Disabil Res. 2021;23(1):360-70.

[43] 43 GÓMEZ, LE; MONSALVE, A; MORÁN, ML; ALCEDO, MA; LOMBARDI, M; SCHALOK, RL. Measurable indicators of CRPD for people with intellectual and developmental disabilities within the quality of life framework. Int J Environ Res Public Health. 2020;17(14):5123.10.3390/ijerph17145123PMC740031832679924

[44] 44 GOMES, JCD. A inclusão social de pessoas com deficiência visual: realidade ou discurso? Dissertação (Mestrado) - Pontifícia Universidade Católica de São Paulo, São Paulo, 2016 29 abr. 2025. https://tede2.pucsp.br/handle/handle/19010.

[45] 45 GOUGH, H; MORRIS, S. Dual diagnosis public policy in a federal system: the Canadian experience. J. Policy Pract. Intellect. Disabil. 2012;9(3):166-74.

[46] 46 GRIGAITĖ, U. Human rights conditions and quality of care in 'Independent Living Homes' for adults, who have intellectual and/or psychosocial disabilities, in Vilnius: analysis of good practice examples, systemic challenges and recommendations for the future. 2017 29 abr. 2025. https://run.unl.pt/entities/publication/29275925-fdfa-455a-bf27-db3052976c3c.

[47] 47 HENRIQUES, ARM. Reensaiar a lucidez: uma análise sobre a incapacidade eleitoral ativa das pessoas com deficiência mental à luz da Constituição. 2023.

[48] 48 HODGSON, D. The educational rights of persons with disabilities: international human rights law and Australian law perspectives. Int. J. Discrim. Law. 2012;12(4):183-220.

[49] 49 HOFFMAN, SJ; SRITHARAN, L; TEJPAR, A. Is the UN Convention on the Rights of Persons with disabilities impacting mental health laws and policies in high-income countries? A case study of implementation in Canada. BMC Int. Health Hum. Rights. 2016;16(1):28.10.1186/s12914-016-0103-1PMC510527427836014

[50] 50 JAFFRÈS, F. Articulation entre politique inclusive et travail protégé des personnes handicapées: le cas suédois. Formation emploi. 2021;(154):177-95.

[51] 51 JONASDOTTIR, SK; EGILSON, SÞP; POLGAR, J; JÓNASDÓTTIR, SK; EGILSON, SÞP; POLGAR, J. Structural factors affecting community mobility for people with mobility impairments in Iceland: a human rights and occupational perspective. J. Occup. Sci. 2021;28(1):59-70.

[52] 52 KELLY, BD. Multi-level protection of rights: India's Mental Healthcare Act, 2017 and the UN Convention on the Rights of Persons with Disabilities (Articles 12 and 14). Int J Law Psychiatry. 2023;91:101937.10.1016/j.ijlp.2023.10193737738687

[53] 53 MAHDANIAN, AA; LAPORTA, M; DREW BOLD, N; FUNK, M; PURAS, D. Human rights in mental healthcare: a review of current global situation. Int Rev Psychiatry. 2023;35(2):150-62.10.1080/09540261.2022.202734837105153

[54] 54 MCCUSKER, P; GILLESPIE, L; DAVIDSON, G; VICARY, S; STONE, K. The UN Convention on the Rights of Persons with Disabilities and social work: evidence for impact? Int J Environ Res Public Health. 2023;20(20).10.3390/ijerph20206927PMC1060659637887665

[55] 55 MARTINS, CD. Deficiência e inclusão em Portugal: as políticas para quem quer ser ouvido. 2018 29 abr. 2025. https://repositorio.ulisboa.pt/entities/publication/074c5208-f6e1-4983-8100-df09f36cb0a1.

[56] 56 MATSUI, R. Employment opportunities of persons with disabilities and the special subsidiary company scheme in Japan. Work. 2013;45(2):261-5.10.3233/WOR-13159223478363

[57] 57 MIRANDA, DHS. Capacidade de exercício das pessoas com deficiência intelectual: institutos jurídicos de proteção e apoio nos ordenamentos português e brasileiro e a Convenção sobre os Direitos das Pessoas com Deficiência. 2024 29 abr. 2025. https://repositorio.grupoautonoma.pt/entities/publication/572a5db6-659d-42a1-b5a3-27122373fa0d.

[58] 58 MOMM, MAL; BARACT, EM; DELGADO, MJG. Trabalho da pessoa com deficiência: harmonização das cotas da deficiência e da aprendizagem a partir da interpretação da Convenção sobre os Direitos da Pessoa com Deficiência. Rev Dir Fundam Relac Trab Soc Emp. 2023;9(2).

[59] 59 MUNOZ-BAELL, IM; ALVAREZ-DARDET, C; RUIZ, MT; FERREIRO-LAGO, E; AROCA-FERNANDEZ, E. Setting the stage for school health-promoting programmes for deaf children in Spain. Health Promot. Internation. 2008;23(4):311-27.10.1093/heapro/dan02618801756

[60] 60 NGWENA, CG. Human right to inclusive education: Exploring a double discourse of inclusive education using South Africa as a case study. Neth. Q. of Hum. Rights. 2013;31(4):473-504.

[61] 61 OLIVEIRA, CB. Aposentadoria da pessoa com deficiência sob a visão dos Direitos Humanos. Dissertação (Mestrado) - Pontifícia Universidade Católica de São Paulo, São Paulo, 2016 29 abr. 2025. https://tede2.pucsp.br/bitstream/handle/19610/2/Carla%20Benedetti%20de%20Oliveira.pdf.

[62] 62 PAGAIME, A. Estudantes com deficiência e as cotas do Sisu: critérios de elegibilidade para ingresso nas universidades federais. Tese (Doutorado) - Universidade de São Paulo, São Paulo, 2022 29 abr. 2025. https://teses.usp.br/teses/disponiveis/48/48137/tde-18012023-104232/publico/ADRIANA_PAGAIME_rev.pdf.

[63] 63 PARMENTER, TR. Rights are necessary but insufficient for the achievement of the full inclusion of people with intellectual and developmental disabilities. Adv Neurodev Disord. 2024;8(1):97-107.

[64] 64 PÉREZ-CURIEL, P; VICENTE, E; MORÁN, ML; GÓMEZ, LE. The right to sexuality, reproductive health, and found a family for people with intellectual disability: a systematic review. Int J Environ Res Public Health. 2023;20(2).10.3390/ijerph20021587PMC986480336674341

[65] 65 PINTO, MTJ. Políticas de proteção social, direitos humanos e deficiência: um estudo comparativo entre Marrocos e a Tunísia. 2020 29 abr. 2025. https://repositorio.ulisboa.pt/entities/publication/d1272500-d61d-4f09-8e56-e4cf255d9a26.

[66] 66 SAIGAL, N; NARAYAN, R. Structural barriers at the workplace for employees with vision and locomotor disabilities in New Delhi, India. Work. 2014;48(3):329-37.10.3233/WOR-14191024939125

[67] 67 SAK, LTB. Inclusão de pessoas com deficiência (PCD) em empresas: uma leitura das práticas de inclusão a partir das considerações finais do comitê sobre os direitos da pessoa com deficiência da ONU sobre o Brasil. 2020 29 abr. 2025. https://bdtd.ibict.br/vufind/Record/PUC_CAMP-5_21c00ba88d158640b55b5c861bea8e97.

[68] 68 SAMPER, MB; FERNÁNDEZ, AC. La inserción de las personas con discapacidad en el empleo público. Análisis jurídico-económico de la situación en la Comunidad de Madrid. CIRIEC-Espana Revista de Economia Publica, Social y Cooperativa. 2017;(91):203-34.

[69] 69 SANTANA, MZ. Políticas públicas de educação inclusiva voltada para estudantes com deficiência na educação superior: o caso da Universidade Federal da Paraíba (UFPB). 2016 29 abr. 2025. https://repositorio.ufpe.br/bitstream/123456789/18049/1/TESE%20FINAL%20Maria%20Z%c3%a9lia%20de%20Santana.pdf.

[70] 70 SANTIAGO, RAM. Trabalhador com deficiência e o emprego apoiado em Portugal e no Brasil. 2016.

[71] 71 SANTOS, RN. Convenção internacional sobre os direitos das pessoas com deficiência no acesso à saúde e à reabilitação: o impacto nas políticas públicas e governança em Cabo Verde. 2024.

[72] 72 SEQUEIRA, BTO. Da inclusão à cidadania: um estudo sobre o grupo de estudos surdos da UCPEL. 2015.

[73] 73 SILVA, AAB. O caminho da inclusão de pessoas com deficiência no mercado de trabalho: onde estamos? 2016.

[74] 74 SILVA, GOD. Acesso e permanência de estudantes surdos no ensino superior: desafios frente à implementação de políticas públicas de inclusão no Instituto Federal de Educação, Ciência e Tecnologia do Espírito Santo. 2023.

[75] 75 SILVEIRA, SVS. Das políticas de inclusão escolar aos projetos políticos pedagógicos da rede comum pública de ensino de Jataí-GO. 2017.

[76] 76 SKEMPES, D; MELVIN, J; VON GROOTE, P; STUCKI, G; BICKENBACH, J. Using concept mapping to develop a human rights based indicator framework to assess country efforts to strengthen rehabilitation provision and policy: The Rehabilitation System Diagnosis and Dialogue framework (RESYST) 16 Studies in Human Society 1605 Policy and Administration 18 Law and Legal Studies 1801 Law. Global Health. 2018;14(1):96.10.1186/s12992-018-0410-5PMC616789130285888

[77] 77 SOUZA, IA. Das capacidades básicas às combinadas como fundamento da dignidade inerente e do direito ao trabalho da pessoa com deficiência. Teoria Jurídica Contemporânea. 2020;5(1):135-57.

[78] 78 SRISUPPAPHON, D; SRIBOONROJ, A; RIEWPAIBOON, W; TANGCHAROENSATHIEN, V. Effective implementation of the UNCRPD by Thailand State Party: challenges and potential remedies. BMC Intern. Health Hum. Rights. 2017;17(1):15.10.1186/s12914-017-0123-5PMC544536728545526

[79] 79 SYMEONIDOU, S. Rights of people with intellectual disability in Cyprus: policies and practices related to greater social and educational inclusion. J. Policy Pract. Intellect. Disabil. 2015;12(2):120-31.

[80] 80 TIBYRIÇÁ, RF. As medidas de apoio para as pessoas com Transtorno do Espectro Autista nas escolas públicas na cidade de São Paulo: análise a partir do direito e da educação brasileira e portuguesa comparados. 2019 29 abr. 2025. https://adelpha-api.mackenzie.br/server/api/core/bitstreams/d99c4bc4-ea61-4bf0-90c0-901d4e936851/content.

[81] 81 TONGSIRI, S; PLOYLEARMSANG, C; HAWSUTISIMA, K; RIEWPAIBOON, W; TANGCHAROENSATHIEN, V. Thaïlande: Modification des domiciles de personnes handicapées physiquement. Bull. WHO. 2017;95(2):140-5.10.2471/BLT.16.178434PMC532793528250515

[82] 82 TRANI, JF; PITZER, KA; VASQUEZ ESCALLON, J; BAKHSHI, P. Access to services from persons with disabilities in Afghanistan: is community based rehabilitation making a difference? Int. J. Environ. Res. Public Health. 2022;19(10):6341.10.3390/ijerph19106341PMC914210335627880

[83] 83 TRIGUEIRO, CS. Políticas afirmativas para pessoas com deficiência e a efetividade do princípio do pleno emprego: o caso dos portadores de visão monocular e surdez unilateral. 2014 29 abr. 2025. https://repositorio.ufpb.br/jspui/bitstream/tede/7784/2/arquivototal.pdf.

[84] 84 VON GRANITZ, H; REINE, I; SONNANDER, K; WINBLAD, U. Do personal assistance activities promote participation for persons with disabilities in Sweden? Disabil Rehabil. 2017;39(24):2512-21.10.1080/09638288.2016.123640527796138

[85] 85 VULCANO, ACS. Direitos humanos, inclusão social e o trabalho da pessoa com deficiência. 2018 29 abr. 2025. http://repositorio.cruzeirodosul.edu.br:8080/jspui/handle/123456789/70.

[86] 86 WALTZ, M; MOL, T; GITTINS, E; SCHIPPERS, A. Disability, access to food and the UN CRPD: Navigating discourses of human rights in the Netherlands. Social Inclusion. 2018;6(1):51-60.

[87] 87 WINKLER, P; KONDRÁTOVÁ, L; KAGSTROM, A; KUCERA, M; PALÁNOVÁ, T; SALOMONOVÁ, M; et al. Adherence to the convention on the rights of people with disabilities in Czech Psychiatric hospitals: a nationwide evaluation study. Health Hum Rights. 2020;22(1):21-33.PMC734843732669786

[88] 88 XAVIER, JCMM. Silêncio e invisibilidade: Iniquidades expressas na comunicação sobre deficiência em campanhas do Ministério da Saúde, entre 1988 e 2020. 2022 29 abr. 2025. https://arca.fiocruz.br/items/ee5f62cd-1bc5-4d99-87e8-e9006ea8e886.

[89] 89 YILMAZ, V. An examination of disability and employment policy in Turkey through the perspectives of disability non-governmental organisations and policy-makers. Disability and Society. 2020;35(5):760-82.

[90] 90 GARCÍA-PIÑA, CA. Forced sterilization of girls and women with disabilities: what health professionals should know to avoid the violation of their rights. Acta Pediatr Mex. 2025;46(2):179-93.

[91] 91 GOULD, M; LEBLOIS, A; CESA BIANCHI, F; MONTENEGRO, V. Convention on the rights of persons with disabilities, assistive technology and information and communication technology requirements: where do we stand on implementation? Disabil. Rehabil. Assist. Technol. 2015;10(4):295-300.10.3109/17483107.2014.97933225364838

[92] 92 MAGNUSSON, L; BICKENBACH, J. Access to human rights for persons using prosthetic and orthotic assistive devices in Sierra Leone. Disability and Rehabilitation. 2020;42(8):1093-100.10.1080/09638288.2018.151526730616394

[93] 93 SILVA, IP. Políticas públicas para inclusão no mercado de trabalho de pessoas com deficiência. Publica-IFRS. 2023;1(1).

[94] 94 WAHYUDI, A; FATHAN, ; KASIYATI, S. Access to justice for persons with disabilities as victims of sexual violence. Inklusi. 2023;10:219-36.

[95] 95 WOLBRING, G; DELORIA, R. Health equity and health inequity of disabled people: a scoping review. Sustainability. 2024;16(16);7143.

[96] 96 BENEDETTI, C; HORVATH JÚNIOR, M. A proteção da pessoa com deficiência como ação afirmativa de inclusão. Rev Int Consinter Dir. 2024;763-84.

[97] 97 GOODLEY, D; RUNSWICK-COLE, K. Problematising policy: conceptions of "child", "disabled" and "parents" in social policy in England. International Journal of Inclusive Education. 2011;15(1):71-85.

[98] 98 NJELESANI, J; CAMERON, C; NJELESANI, D; JAMALI, M; ANDRION, JJD; MUNTHALI, A. Recognizing the rights of children with disabilities in Malawi. Disabil Soc. 2024;40(7):1853-71.

[99] 99 SHIKAKO, K; LENRUCHA, R; HUNT, M; JODOIN-PILON, S; CHANDRA, A; KATALIFOS, A; et al. Children with disabilities in Canada during the COVID-19 pandemic: an analysis of COVID-19 policies through a disability rights lens. Children. 2023;10(6):942.10.3390/children10060942PMC1029689137371173

[100] 100 FURGALSKA, M; DE LONDRAS, F. Towards a rights-based approach for disabled women's access to abortion. Med. Law. Rev. 2024;32(4):486-504.10.1093/medlaw/fwae026PMC1158652939018550

[101] 101 LEE, K; DEVINE, A; MARCO, MJ; ZAYAS, J; GILL-ATKINSON, L; VAUGHAN, C. Sexual and reproductive health services for women with disability: a qualitative study with service providers in the Philippines. BMC Women's Health. 2015;15(1):87.10.1186/s12905-015-0244-8PMC460655626466892

[102] 102 RADE, BK; TAMIRU, AT; AYALEM, GL; TAYE, EB; MELKIE, M; ABERA, A; et al. Prevalence and factors associated with sexual and reproductive health services use among reproductive age women with disabilities: a community-based cross-sectional study. BMC Womens Health. 2023;23(1):215.10.1186/s12905-023-02373-5PMC1015529537131161

[103] 103 COE, S. D'autres enseignements concrets tirés de cinq projets de développement incluant les personnes handicapées. Development in Practice. 2012;22(3):400-8.

[104] 104 EYRAUD, B. A human rights approach to disability? Applying the spiral model to the French adoption of the Convention on the Rights of Persons with Disabilities. Droit Soc. 2023;113(1):55-71.

[105] 105 COLON-CABRERA, D; SHARMA, S; WARREN, N; SAKELLARIOU, D. Examining the role of government in shaping disability inclusiveness around COVID-19: a framework analysis of Australian guidelines. International Journal for Equity in Health. 2021;20:1-11.10.1186/s12939-021-01506-2PMC828374734271933

[106] 106 GRANGAARD, S; HEDVALL, PO; LID, IM. Universal design and accessibility as an act or a state: a comparison of policies in Norway, Sweden, and Denmark. Stud Health Technol Inform. 2024;320:2-9.10.3233/SHTI24097639560218

[107] 107 BARRERA JUAREZ, YF. Inclusão de pessoas com deficiência no ensino superior: um panorama Brasil-México. 2020 29 abr. 2025. https://repositorio.utfpr.edu.br/jspui/bitstream/1/4982/2/inclusaodeficienciabrasilmexico.pdf.

[108] 108 BICKENBACH, JE. Monitoring the United Nations convention on the rights of persons with disabilities: data and the international classification of functioning, disability and health. BMC Public Health. 2011;11(Suppl 4):S8.10.1186/1471-2458-11-S4-S8PMC310422121624194

[109] 109 FERRI, D; FAVALLI, S. Web accessibility for people with disabilities in the European Union: paving the road to social inclusion. Societies. 2018;8(2):40.

[110] 110 FERRI, D; GIANNOUMIS, GA. A Revaluation of the cultural dimension of disability policy in the European Union: the impact of digitization and web accessibility. Behav Sci Law. 2014;32(1):33-51.10.1002/bsl.210224676850

[111] 111 GARG, A; SKEMPES, D; BICKENBACH, J. Legal and regulatory approaches to rehabilitation planning: a concise overview of current laws and policies addressing access to rehabilitation in five European countries. Int. J. Environ. Res. Public Health. 2020;17(12):1.10.3390/ijerph17124363PMC734608832570746

[112] 112 LORD, JE; STEIN, MA; FIALA-BUTORA, J. Facilitating an equal right to vote for persons with disabilities. J. Hum. Rights Pract. 2014;6(1):115-39.

[113] 113 MARINHO, MG. Acesso e permanência de pessoas com deficiência no mercado de trabalho: a alternativa do emprego apoiado. Revista Interinstitucional Brasileira de Terapia Ocupacional. 2019;3(2):260-76.

[114] 114 MAXWELL, N. Self-determination and community inclusion: exploring policy reform in day services for adults with intellectual and developmental disabilities in the Republic of Ireland. J Policy Pract Intellect Disabil. 2025;22(1):e70003.

[115] 115 MCCARTHY, TS. Regulating restraint and seclusion in Australian government schools: a comparative human rights analysis. QUT Law Review. 2019;18(2):194.

[116] 116 MIRA-ALADRÉN, M; MARTÍN-PEÑA, J; GIL-LACRUZ, M. Evaluation of social interventions with people with disabilities: a systematic literature review. Soc Work Res. 2024;48(2):126-39.

[117] 117 MORGAN, C. The experiences of disabled people in the United Arab Emirates: barriers to participation in higher education and employment. Disabil Soc. 2023;38(3):421-44.

[118] 118 QUEIROZ, IFR. Atenção primária à saúde das pessoas com deficiências. 2023 29 abr. 2025. https://repositorio.ufc.br/bitstream/riufc/77657/7/2022_dis_ifrqueiroz.pdf.

[119] 119 RAMOS, SG. Educação inclusiva: uma reflexão sobre a política social da pessoa com deficiência. 2015 29 abr. 2025. https://tede2.pucgoias.edu.br/bitstream/tede/2193/1/SANDRA%20GOMES%20RAMOS.pdf.

[120] 120 RESENDE, EA. O direito fundamental ao trabalho da pessoa com deficiência no Brasil: caminhos para a eficácia social. 2016 29 abr. 2025. https://ri.ufs.br/bitstream/riufs/4385/1/EMERSON_ALBUQUERQUE_RESENDE.pdf.

[121] 121 AMORIM, MM. Direito a educação superior para pessoas com deficiência no Brasil e Uruguai: estudo comparativo no decênio da inclusão -2006-2016. 2019 29 abr. 2025. https://repositorio.unb.br/bitstream/10482/36820/1/2019_ManoelMendesAmorim.pdf.

[122] 122 ANDRADE, RM. Práticas inclusivas orientadas ao estudante com deficiência intelectual no ensino profissional técnico de nível médio no campus Boituva - IFSP. 2019 29 abr. 2025. https://repositorio.cruzeirodosul.edu.br/items/6bb8c466-4a74-4180-b6c2-d7a904f65da7.

[123] 123 CABRAL, RLG; OLIVEIRA, RRN; REIS, ULS. A implementação do direito à educação superior das pessoas com deficiência no Brasil: avanços e desafios pendentes na agenda de concretização da cidadania. Pensar. 2022;27(4):1-14.

[124] 124 CHAGAS, PHRS. O direito à educação para as pessoas com deficiência nos atos normativos produzidos pelo Conselho Nacional de Educação (2008-2019). 2020 29 abr. 2025. https://repositorio.unb.br/bitstream/10482/41323/1/2020_Patr%c3%adciaHelenaRibeirodeSouzaChagas.pdf.

[125] 125 CISTERNAS, DS; AGUILA, MF. Citizen participation of people with disabilities and intersectoral public policies offered by the state in the region of Magallanes, Chile. Revista Del Clad Reforma Y Democracia. 2020;(77):187-214.

[126] 126 CURITIBA, JA. Educação especial no Brasil e a Lei Brasileira de Inclusão: aproximações e afastamentos. 2020 29 abr. 2025. https://repositorio.unicamp.br/Busca/Download?codigoArquivo=449208.

[127] 127 DURHAM, J; SYCHAREUN, V; RODNEY, A; GOUDA, HN; RICHARDS, N; RAMPATIGE, R; et al. Health information systems and disability in the Lao PDR: a qualitative study. Int. J. Health Plann. Manage. 2016;31(4):446-58.10.1002/hpm.231926490708

[128] 128 GARRIDO-CUMBRERA, M; CHACÓN-GARCÍA, J; SCHÄDLER, J. The Convention on the Rights of Persons With Disabilities Un: analysis of the implementation in Andalucía (Spain) of Article 33. Rev. Chil. Ter. Ocup. 2016;16(2):119.

[129] 129 MORGAN, C. The experiences of disabled people in the United Arab Emirates: Barriers to participation in higher education and employment. Disability and Society. 2023;38(3):421-44.

[130] 130 MURPHY, M; THOMPSON, S; DOYLE, DM; FERRI, D. Inclusive education and the law in Ireland. Int. J. Law Context. 2022;19(2):1-21.

[131] 131 REVILLARD, A. Vulnerable rights: the incomplete realization of disability social rights in France. Social Sciences. 2018;7(6):88.

[132] 132 ELEKANACHI, RU; SHIKAKO, K; SNIDER, L; DAHAN-OLIEL, N. A portrait of the rights of children with disabilities in Nigeria: a policy review. Int. J. Environ. Res. Public Health. 2023;20(21):6996.10.3390/ijerph20216996PMC1065076237947554

[133] 133 GRIGAITĖ, U; LEVICKAITĖ, K; JUODKAITĖ, D; GOŠTAUTAITĖ-MIDTTUN, N. Promoting human rights-based deinstitutionalization in Lithuania by applying the WHO's QualityRights assessments. Int. J. Qual. Health Care. 2025;37(1):118.10.1093/intqhc/mzae11839739416

[134] 134 VAN HOUT, MC; KAIMA, R; MHANGO, V; KEWLEY, S; MARINIELLO, T. Judiciarisation of the mentally ill and/or mentally incapacitated in the Malawi criminal justice system: gaps and flaws of human rights protection. Forensic Sci. Int. Mind Law. 2023;4:100121.

[135] 135 SHIKAKO, K; LENRUCHA, R; HUNT, M; JODOIN, S; ELSABBAGH, M; HUDON, A; et al. How did governments address the needs of people with disabilities during the COVID-19 pandemic? An analysis of 14 countries' policies based on the UN Convention on the Rights of Persons with Disabilities. Int. J. Health Policy Manag. 2023;12(1):7111.10.34172/ijhpm.2023.7111PMC1042565637579394

[136] 136 STAVERT, J; MCKAY, C. Scotland, the Convention on the Rights of Persons with Disabilities and repurposing of mental health and capacity law. Hum Rights Law Rev. 2024;25(2).

[137] 137 YANG, DWJ. Disability discrimination in the provision of health insurance: Article 25(e) of the UN Convention on the Rights of Persons with Disabilities. Int. J. Discrim. Law. 2024;24(1):48-66.

[138] 138 SCHERER, N; BANDA-CHALWE, M; CHANSA-KABALI, T; NSEIBO, K; SEKETI, QE; MCKENZIE, J; et al. Disability research in Zambia: a scoping review. Scand. J. Disabil. Res. 2024;26(1):44-66.

[139] 139 SCHIDEL, R. Universal enfranchisement for citizens with cognitive disabilities: a moral-status argument. Crit. Rev. Int. Soc. Polit. Philos. 2023;26(5):658-79.

[140] 140 SULEIMAN, NM; MAJEED, ASA; ZUHAIR, M; JASIM, BAA; TURCHYN, M. Global impact and intersectionality: a comprehensive analysis of the CRPD's implementation and the future of disability rights in international law. J. Ecohumanism. 2024;3(5):467-80.

[141] 141 MORALES, JG. Progresividad en el acceso al trabajo de las mujeres con discapacidad en Colombia. Vniversitas. 2022;71:1-14.

[142] 142 SILVA, LMVD. Conceitos, abordagens e estratégias para a avaliação em saúde. In: HARTZ, ZMA; SILVA, LMV (org). Avaliação em saúde: dos modelos teóricos à prática na avaliação de programas e sistemas de saúde. Rio de Janeiro: Editora Fiocruz, 2005. p. 15-39.

[143] 143 LASCOUMES, P; LE GALÈS, P (org). Sociologia da Ação Pública. Editora da Universidade Federal de Alagoas, 2012 12 set. 2024. https://sciencespo.hal.science/hal-02100045.

[144] 144 World Health Organization. Relatório mundial sobre a deficiência. São Paulo: SEDPcD, 2012. https://iris.who.int/server/api/core/bitstreams/6ec54f7c-5586-4e2e-bb13-e960fa979a81/content.

[145] 145 WALSH, PN. Health indicators and intellectual disability. Curr. Opin. Psychiatry. 2008;21(5):474-8.10.1097/YCO.0b013e3283065bc618650690

[146] 146 FURTADO, JP; ONOCKO-CAMPOS, RT; MOREIRA, MIB; TRAPÉ, TL. A elaboração participativa de indicadores para a avaliação em saúde mental. Cad. Saúde Pública. 2013;29:102-10.10.1590/s0102-311x201300010001223370029

[147] 147 Organização Mundial da Saúde. Classificação Internacional de Funcionalidade, Incapacidade e Saúde. São Paulo: Editora da Universidade de São Paulo, 2019.
